# Terbium-161 for PSMA-targeted radionuclide therapy of prostate cancer

**DOI:** 10.1007/s00259-019-04345-0

**Published:** 2019-05-27

**Authors:** Cristina Müller, Christoph A. Umbricht, Nadezda Gracheva, Viviane J. Tschan, Giovanni Pellegrini, Peter Bernhardt, Jan Rijn Zeevaart, Ulli Köster, Roger Schibli, Nicholas P. van der Meulen

**Affiliations:** 1 Center for Radiopharmaceutical Sciences ETH-PSI-USZ, Paul Scherrer Institute, 5232 Villigen, Switzerland; 2 Laboratory for Animal Model Pathology, Institute of Veterinary Pathology, Vetsuisse Faculty, University of Zurich, 8057 Zurich, Switzerland; 3 Department of Radiation Physics, Institution of Clinical Science, Sahlgrenska Academy, University of Gothenburg, 413 45 Gothenburg, Sweden; 4 Radiochemistry, South African Nuclear Energy Corporation (Necsa), Brits, 0240 South Africa; 5 Institut Laue-Langevin, 38042 Grenoble, France; 6 Department of Chemistry and Applied Biosciences, ETH Zurich, 8093 Zurich, Switzerland; 7 Laboratory of Radiochemistry, Paul Scherrer Institute, 5232 Villigen, Switzerland

**Keywords:** ^161^Tb, Auger electrons, Prostate cancer, PSMA ligands, Radioligand therapy

## Abstract

**Purpose:**

The prostate-specific membrane antigen (PSMA) has emerged as an interesting target for radionuclide therapy of metastasized castration-resistant prostate cancer (mCRPC). The aim of this study was to investigate ^161^Tb (T_1/2_ = 6.89 days; Eβ^-^uperscript>_av_ = 154 keV) in combination with PSMA-617 as a potentially more effective therapeutic alternative to ^177^Lu-PSMA-617, due to the abundant co-emission of conversion and Auger electrons, resulting in an improved absorbed dose profile.

**Methods:**

^161^Tb was used for the radiolabeling of PSMA-617 at high specific activities up to 100 MBq/nmol. ^161^Tb-PSMA-617 was tested in vitro and in tumor-bearing mice to confirm equal properties, as previously determined for ^177^Lu-PSMA-617. The effects of ^161^Tb-PSMA-617 and ^177^Lu-PSMA-617 on cell viability (MTT assay) and survival (clonogenic assay) were compared in vitro using PSMA-positive PC-3 PIP tumor cells. ^161^Tb-PSMA-617 was further investigated in therapy studies using PC-3 PIP tumor-bearing mice.

**Results:**

^161^Tb-PSMA-617 and ^177^Lu-PSMA-617 displayed equal in-vitro properties and tissue distribution profiles in tumor-bearing mice. The viability and survival of PC-3 PIP tumor cells were more reduced when exposed to ^161^Tb-PSMA-617 as compared to the effect obtained with the same activities of ^177^Lu-PSMA-617 over the whole investigated concentration range. Treatment of mice with ^161^Tb-PSMA-617 (5.0 MBq/mouse and 10 MBq/mouse, respectively) resulted in an activity-dependent increase of the median survival (36 vs 65 days) compared to untreated control animals (19 days). Therapy studies to compare the effects of ^161^Tb-PSMA-617 and ^177^Lu-PSMA-617 indicated the anticipated superiority of ^161^Tb over ^177^Lu.

**Conclusion:**

^161^Tb-PSMA-617 showed superior in-vitro and in-vivo results as compared to ^177^Lu-PSMA-617, confirming theoretical dose calculations that indicate an additive therapeutic effect of conversion and Auger electrons in the case of ^161^Tb. These data warrant more preclinical research for in-depth investigations of the proposed concept, and present a basis for future clinical translation of ^161^Tb-PSMA-617 for the treatment of mCRPC.

**Electronic supplementary material:**

The online version of this article (10.1007/s00259-019-04345-0) contains supplementary material, which is available to authorized users.

## Introduction

The prostate-specific membrane antigen (PSMA) is a cell-surface glycoprotein that is expressed in normal prostate tissue and overexpressed in prostate cancer [1, 2]. There are indications that the expression level of PSMA correlates with the stage of the disease and the risk of disease progression [3, 4]. PSMA is, therefore, an interesting target to use for radionuclide therapy of metastasized castration-resistant prostate cancer (mCRPC) [5–8]. The topic of PSMA targeting became popular with the development of small-molecule-based radioligands [9]. Initial compounds were designed for radioiodination, suitable for nuclear imaging, and the first to be used therapeutically in patients [10]. Subsequently, PSMA ligands were developed with a chelator to allow their use in combination with radiometals for both imaging and therapeutic purposes [5, 8, 11]. PSMA-617 and PSMA I&T, equipped with a DOTA and DOTAGA chelator, respectively, have been used for targeted radionuclide therapy of mCRPC in clinics [7, 12, 13]. For this purpose, they were mostly labeled with ^177^Lu (T_1/2_ = 6.65 d; Eβ^-^_av_ = 134 keV; Eγ = 113 keV, I = 6.17%, Eγ = 208 keV, I = 10.36%), which is currently the most-often applied radiometal for therapeutic purposes in the clinics [14]. In specific cases, ^225^Ac-PSMA-617 was employed for the treatment of patients at end-stage without further treatment options [15–17]. ^225^Ac decays with a half-life of 10 days, emitting several α- and β¯-particles while decaying via a sequence of radioactive daughter nuclides [18]. Although the results obtained with ^225^Ac-PSMA-617 were impressive, undesired side effects — referring to irreversible damage of salivary and lacrimal glands — have been reported [17]. The question arises, therefore, whether alternative radiometals could be used for targeted radionuclide therapy of mCRPC which would be potentially more powerful than the currently-employed ^177^Lu, without causing additional side-effects.

In this work, we investigated ^161^Tb, a recently-introduced radiolanthanide for therapeutic applications [19]. ^161^Tb decays with a half-life of 6.89 days to stable ^161^Dy, while emitting β¯-particles (Eβ^-^_av_ = 154 keV) suitable for therapeutic purposes and γ-radiation (Eγ = 49 keV, I = 17.0%; Eγ = 75 keV, I = 10.2%) useful for SPECT imaging. In this regard, ^161^Tb closely resembles ^177^Lu, even though the emitted γ-radiation is of lower energy. ^161^Tb also emits a substantial number of low-energy conversion and Auger electrons, which makes this radionuclide exceptionally interesting for the treatment of disseminated cancers with multiple metastases ranging from a single cell (diameter: ~10 μm) to micro cell clusters (diameter: < 1 mm) [20]. Monte Carlo simulations performed by Hindié et al. to assess the dose delivered to 10-μm spheres revealed a 3.5-fold increased value when using ^161^Tb as compared to ^177^Lu [21]. In larger tumors (diameter > 10 mm), the emitted electron energy from ^161^Tb and ^177^Lu respectively is almost entirely absorbed, resulting in a 1.3-fold higher absorbed electron energy fraction per decay for ^161^Tb (total electron emission of 197 keV/decay) compared to ^177^Lu (147 keV/decay), making ^161^Tb more potent than ^177^Lu [21]. An additional advantage of ^161^Tb over ^177^Lu may be the existence of diagnostic counterparts, including ^152^Tb (T_1/2_ = 17.5 h; Eβ^+^_av_ = 1140 keV, I = 20.3%) and ^155^Tb (T_1/2_ = 5.32 d; Eγ = 87 keV, 32.0%, 105 keV, I = 25.1%) for PET and SPECT imaging respectively, potentially enabling pre-therapeutic dosimetry with chemically identical radiopharmaceuticals [22–25]. The results of theoretical calculations performed by Champion and co-workers also indicate that ^161^Tb outperforms other clinically employed (^177^Lu, ^90^Y) and non-standard therapeutic radionuclides (^47^Sc, ^67^Cu) with regard to the dose delivery to small lesions [21, 26].

The production of ^161^Tb via the ^160^Gd(n,γ)^161^Gd → ^161^Tb nuclear reaction was previously reported by Lehenberger et al. [19]. At the Paul Scherrer Institute (PSI), the method of processing Gd targets irradiated in high neutron flux reactors (RHF, Institut Laue-Langevin, Grenoble, France or SAFARI-1, Necsa, Pelindaba, South Africa) or at a spallation neutron source (SINQ, PSI, Switzerland) was implemented some years ago [22]. The chemical separation of ^161^Tb from the target material has since been further developed and optimized at PSI.

The topic of the present study was to investigate ^161^Tb with regard to its application for radionuclide therapy. ^161^Tb was, therefore, used to label PSMA-617 to enable preclinical comparison with ^177^Lu-PSMA-617. The in-vitro experiments and biodistribution studies in PC-3 PIP/flu tumor-bearing mice were performed to confirm equal chemical and pharmacokinetic properties of ^161^Tb-PSMA-617 and ^177^Lu-PSMA-617 respectively. Importantly, the effect of ^161^Tb-PSMA-617 was compared to that obtained with ^177^Lu-PSMA-617 by means of in-vitro cell viability and survival assays, and the therapeutic effect of ^161^Tb-PSMA-617 was shown in vivo using tumor-bearing mice.

## Materials and methods

### Production and chemical separation of ^161^Tb

^161^Tb was produced as previously reported [22]. Enriched ^160^Gd targets were irradiated over a period of 1–2 weeks at the SAFARI-1 reactor at Necsa, Pelindaba, South Africa, or at the RHF at Institut Laue–Langevin, Grenoble, France. In some cases, 3-week irradiations were performed at the spallation-induced neutron source SINQ, PSI, Switzerland. ^161^Tb was chemically separated from the Gd target material and impurities by cation exchange chromatography, using an optimized method of the previously-published process (Supplementary Material) [19, 22].

### Preparation and in-vitro evaluation of ^161^Tb-PSMA-617

The radiolabeling of PSMA-617 (Advanced Biochemical Compounds, ABX GmbH, Radeberg, Germany) with ^161^Tb was performed under standard labeling conditions (Supplementary material). The stability of ^161^Tb-PSMA-617, incubated in saline (250 MBq/500 μL), was investigated over a period of 24 h at room temperature (Supplementary material). The *n*-octanol/PBS distribution coefficient (logD) was determined for ^161^Tb-PSMA-617 (Supplementary material). All of these experiments were performed as previously reported for ^177^Lu-PSMA-617 [27].

### Tumor cell uptake and internalization studies

Uptake and internalization studies of ^161^Tb-PSMA-617 and ^177^Lu-PSMA-617 were performed, as previously reported, using PSMA-positive PC-3 PIP and PSMA-negative PC-3 flu tumor cells (provided by Prof. Dr. Martin Pomper; John Hopkins University, Baltimore, USA) (Supplementary material) [27].

### Cell viability assay (MTT assay) and cell survival assay (clonogenic assay)

Tumor cell viability of PC-3 PIP/flu tumor cells upon exposure to ^161^Tb-PSMA-617 and ^177^Lu-PSMA-617 (0.01–20 MBq/mL) was assessed using a 3-(4,5-dimethylthiazol-2-yl)-2,5-diphenyltetrazolium bromide (MTT) assay, as described by Mosmann [28], and performed according to a previously-reported procedure [29]. The survival of PC-3 PIP/flu tumor cells upon exposure to ^161^Tb-PSMA-617 and ^177^Lu-PSMA-617 (0.01–10 MBq/mL) was determined using the clonogenic assay, as described by Franken et al. [30], and performed according to a previously-reported procedure [31]. The detailed methods of these studies, including dosimetric calculations, are described in the Supplementary material. The results were analyzed for statistical significance by a two-way ANOVA with Sidak’s multiple comparison post-test using Graph Pad Prism (version 7).

### In-vivo studies

In-vivo experiments were approved by the local veterinarian department and conducted in accordance with the Swiss law of animal protection. Athymic nude BALB/c mice were obtained from Charles River Laboratories (Sulzfeld, Germany) at the age of 5–6 weeks. Mice were subcutaneously inoculated with PC-3 PIP tumor cells (6 × 10^6^ cells in 100 μL Hank’s balanced salt solution (HBSS) with Ca^2+^/Mg^2+^) and PSMA-negative PC-3 flu tumor cells (5 × 10^6^ cells in 100 μL HBSS with Ca^2+^/Mg^2+^) on the right and left shoulder, respectively, for biodistribution and SPECT imaging studies. Therapy studies were performed with mice inoculated with PC-3 PIP cells (4 × 10^6^ cells in HBSS with Ca^2+^/Mg^2+^) on the right shoulder (Supplementary material).

### Biodistribution studies

Biodistribution studies were performed 12–14 days after tumor cell inoculation when the tumor xenografts reached an average tumor volume of about ~50–200 mm^3^ (Supplementary material). PSMA-617 was labeled with ^161^Tb at a specific activity of 5.0 MBq/nmol and diluted in saline. Tumor-bearing mice were intravenously injected with ^161^Tb-PSMA-617 (5.0 MBq, 1 nmol, 100 μL). The mice were sacrificed at 1 h, 4 h, 24 h, 48 h, or 96 h post injection (p.i.) and selected tissues and organs were collected, weighed, and measured using a γ-counter (Perkin Elmer, Wallac Wizard 1480). Groups of 3–5 mice were sacrificed at each time point. The results were decay-corrected and listed as percentage of the injected activity per gram of tissue mass (% IA/g). Data are presented as the average ± standard deviation (SD).

The data were compared with those previously obtained for ^177^Lu-PSMA-617 [27] and analyzed for significance using a one-way ANOVA with Tukey’s multiple comparison post-test using GraphPad Prism software (version 7). A *p*-value of < 0.05 was considered statistically significant.

### Dosimetry estimations

The mean specific absorbed doses (Gy/MBq) to the tumor xenografts and the kidneys were estimated for ^161^Tb-PSMA-617 and ^177^Lu-PSMA-617 (Supplementary material). The tissue distribution profile of ^161^Tb-PSMA-617 was considered as equal to the previously-determined biodistribution data of ^177^Lu-PSMA-617 [27, 32]. The [% IA/g] values were converted to non-decay corrected values using the respective half-lives of the radionuclides to obtain time-integrated activity to infinity. The mean absorbed energy per decay to cells in the cell viability study was calculated using Monte Carlo simulations with PENELOPE-2014 [33].

### SPECT/CT imaging studies

In a separate study, SPECT/CT experiments were performed 12–14 days after tumor cell inoculation using a dedicated small-animal SPECT/CT camera (NanoSPECT/CT^TM^, Mediso Medical Imaging Systems, Budapest, Hungary) as previously reported (Supplementary material) [34]. ^161^Tb-PSMA-617 (~25 MBq/nmol) was diluted in saline for injection. Scans were acquired at 1 h, 4 h, and 24 h after injection of the radioligands (~25 MBq, 1 nmol, 100 μL). During the in-vivo scans, mice were anesthetized using a mixture of Isoflurane and oxygen.

### Therapy study

Three groups of mice (*n* = 6) were injected with only saline, ^161^Tb-PSMA-617 (5.0 MBq; 1 nmol/mouse), or ^161^Tb-PSMA-617 (10 MBq; 1 nmol/mouse) at Day 0 of the therapy, 6 days after PC-3 PIP tumor cell inoculation (Table 1). The mice were monitored by measuring body weights and the tumor sizes every other day over 12 weeks. Mice were euthanized when pre-defined endpoint criteria were reached, or when the study was terminated at Day 84 (Supplementary material). The relative body weight (RBW) was defined as [BW_x_/ BW_0_], where BW_x_ is the body weight in gram at a given day x, and BW_0_ the body weight in grams at Day 0. The tumor dimension was determined by measuring the longest tumor axis (L) and its perpendicular axis (W) with a digital caliper. The tumor volume (V) was calculated according to the eq. [V = 0.5 * (L * W^2^)]. The relative tumor volume (RTV) was defined as [TV_x_/TV_0_], where TV_x_ is the tumor volume in mm^3^ at a given day x, and TV_0_ the tumor volume in mm^3^ at Day 0.

**Table 1:** Design of the tumor therapy study

Treatment groups (*n* = 6)	Injected activity^1^	Calculated absorbed dose to tumors	Tumor volume	Body weight
			Day 0	Day 0
	(MBq)	(Gy)	(mm^3^)	(g)
Saline	–	–	80 ± 17	17 ± 0.4
^161^Tb-PSMA-617	5.0	27	74 ± 28	17 ± 1.2
^161^Tb-PSMA-617	10	54	88 ± 27	18 ± 1.0*

^1^The quantity of activity of the injection solutions for each group was confirmed by counting an injection sample (100 μL) using the dose calibrator.

^*^The average body weight of mice injected with 10 MBq ^161^Tb-PSMA-617 was significantly higher than the average body weight of mice injected with 5.0 MBq ^161^Tb-PSMA-617 (*p* < 0.05).

### Assessment of therapy study

The efficacy of the radionuclide therapy was assessed by the tumor growth delay (TGD_x_), which was calculated as the time required for the tumor volume to increase x-fold over the initial volume at Day 0. The tumor growth delay index [TGDI_x_ = TGD_x_(T)/TGD_x_(C)] was calculated as the TGD_x_ ratio of treated mice (T) over control mice (C) for a 2-fold (x = 2, TGD_2_) and 5-fold (x = 5, TGD_5_) increase of the initial tumor volume. Statistical analysis was performed by a one-way ANOVA with Tukey’s multiple comparison post-test using GraphPad Prism software (version 7). A value of *p* < 0.05 was considered statistically significant. The median survival was calculated by Kaplan–Meier curves using GraphPad Prism software (version 7).

Potential early side-effects related to the exposure to radiation were evaluated by the assessment of absolute and relative (to body and to brain) organ weights, selected clinical chemistry plasma parameters including creatinine (CRE), blood urea nitrogen (BUN), alkaline phosphatase (ALP), total bilirubin (TBIL), and albumin (ALB), as well as via histological analysis of bone marrow and salivary glands. The data were analyzed for statistical significance (Supplementary material).

### Additional in-vivo investigations using ^161^Tb-PSMA-617

Additional investigations performed in mice that received ^161^Tb-PSMA-617 or ^177^Lu-PSMA-617 (2.5 MBq/mouse, 5.0 MBq/mouse or 10 MBq/mouse) 2 days after PC-3 PIP tumor cell inoculation are reported in the Supplementary material.

## Results

### Production of ^161^Tb

No-carrier-added ^161^Tb was produced at high activities of 6–20 GBq (end of irradiation) depending on the irradiation parameters (neutron flux, irradiation time, and mass of target material). The chemical separation resulted in a radionuclidically pure (^160^Tb < 0.007%) product of high radiochemical purity comparable to commercial, no-carrier-added ^177^Lu (Supplementary material, Table S1). ^161^Tb was made available at a high-activity concentration (10–20 MBq/μL) in Suprapur^TM^ HCl (0.05 M) to be used directly for radiolabeling of PSMA-617.

### Radiolabeling, stability and in-vitro properties

Radiolabeling of PSMA-617 with ^161^Tb was achieved at specific activities up to 100 MBq/nmol at a radiochemical purity of ≥98% (Supplementary material, Fig. S1). ^161^Tb-PSMA-617 (50 MBq/nmol; 250 MBq/500 μL) was stable over at least 1 h (> 98%), but showed radiolytic degradation when incubated for longer time periods. In the presence of l-ascorbic acid, ^161^Tb-PSMA-617 was stable up to 24 h (≥ 98%) and did not show any signs of radiolytic degradation (Supplementary material, Fig. S2). The determination of the *n*-octanol/PBS distribution coefficient (logD value) of ^161^Tb-PSMA-617 resulted in a value of −3.9 ± 0.1 (Supplementary material).

### Internalization studies

The PC-3 PIP tumor cell uptake of ^161^Tb-PSMA-617 (47–54%) and the internalized fraction (8–11%) after 2–4 h incubation was comparable to the data obtained with ^177^Lu-PSMA-617 (49–58% and 9–12%, respectively). The uptake in PC-3 flu tumor cells was < 0.5% for both ^161^Tb-PSMA-617 and ^177^Lu-PSMA-617, respectively (Supplementary material, Fig. S3).

### In-vitro tumor-cell viability and survival

**Fig. 1:**
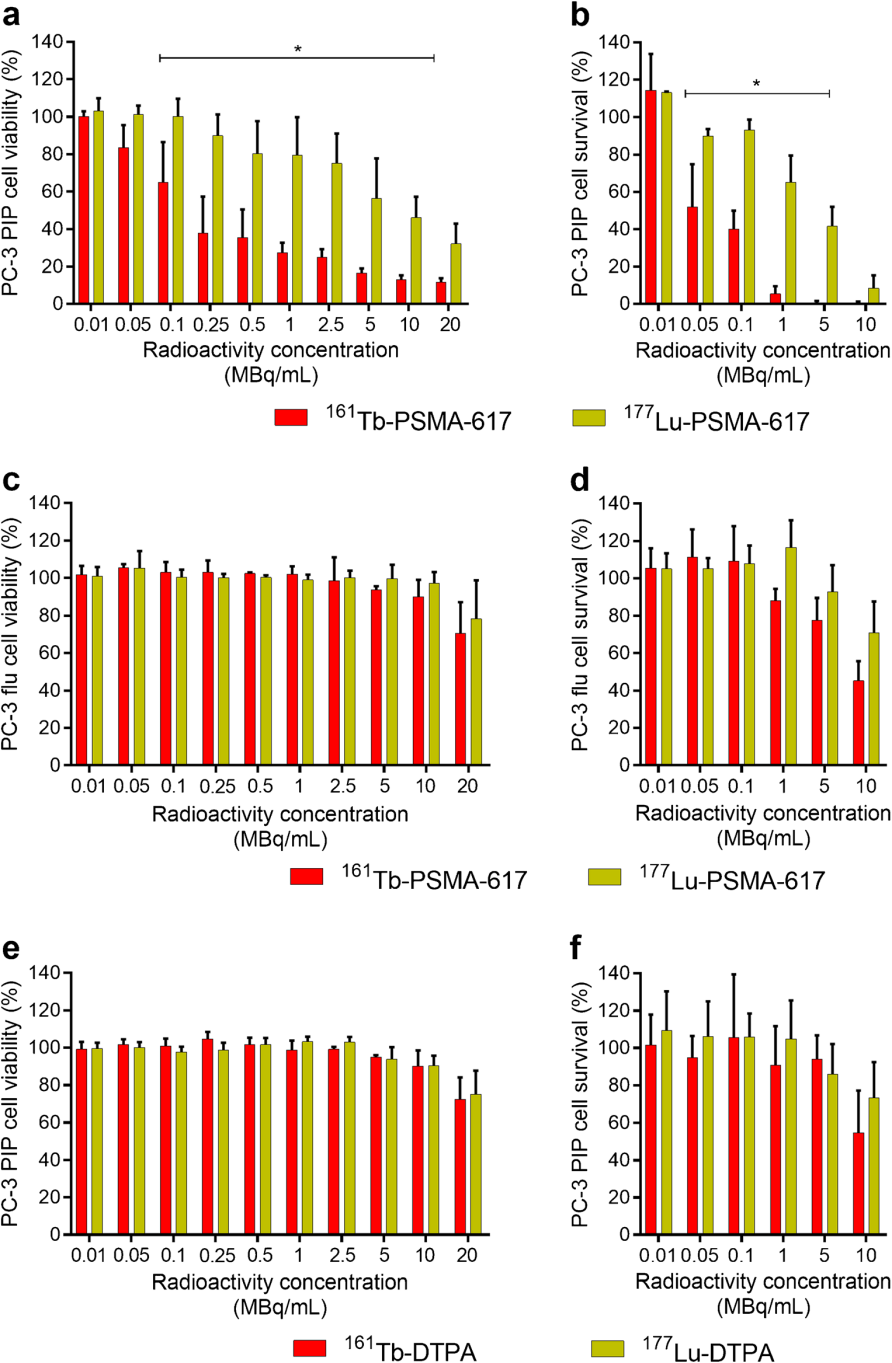
Results of the PC-3 PIP/flu tumor (**a/c/e**) cell viability and (**b/d/f**) survival studies. **a**, **b** Percentage of PC-3 PIP tumor cell viability and survival using ^161^Tb-PSMA-617 and ^177^Lu-PSMA-617 compared to untreated control cells (set to 100% viability; average ± SD). **c**, **d** Percentage of PC-3 flu tumor cell viability using ^161^Tb-PSMA-617 and ^177^Lu-PSMA-617 compared to untreated control cells (set to 100% viability; average ± SD). **e**, **f** Percentage of PC-3 PIP tumor cell viability using ^161^Tb-DTPA and ^177^Lu-DTPA compared to untreated control cells (set to 100% viability; average ± SD). * indicates the range where significantly different data was obtained for ^161^Tb-PSMA-617 and ^177^Lu-PSMA-617 (*p* < 0.05).

The reduction of viability and survival of PC-3 PIP tumor cells after exposure to ^161^Tb-PSMA-617 and ^177^Lu-PSMA-617 correlated with the applied activity concentration. ^161^Tb-PSMA-617 was significantly more effective in reducing the tumor-cell viability (determined by MTT assays) and survival (determined by clonogenic assays) as compared to ^177^Lu-PSMA-617 when applied at activity concentrations in the range of 0.1–10 MBq/mL (*p* < 0.05) and 0.05–5.0 MBq/mL (*p* < 0.05) respectively (Fig. 1a/b). Under the given experimental conditions, the mean absorbed energy to tumor cells in MTT assays was calculated to be 3.2–4.2-fold higher for ^161^Tb than for ^177^Lu. Lower values reflect the situation for cell monolayers, whereas the higher value refers to the “single-cell situation” which was more the case during the treatment, particularly in the setting of the clonogenic assay where the cell number per well was low. The viability of PSMA-negative PC-3 flu cells was not affected when the radioligands were applied at concentrations of up to 10 MBq/mL. Only a slight reduction that was equal for both radioligands (*p* > 0.05) was detected at the highest concentration (20 MBq/mL). The survival of PC-3 flu cells was, however, affected at radioligand concentrations of 1 MBq/mL and higher, with a tendency of a more pronounced effect from ^161^Tb-PSMA-617 (*p* > 0.05) (Fig. 1c/d). The viability and survival of PC-3 PIP tumor cells exposed to ^161^Tb-DPTA and ^177^Lu-DTPA were not affected, and showed only a marginal reduction at higher radioligand concentration, which was equal for both radionuclide complexes (*p* > 0.05) (Fig. 1e/f).

### Biodistribution studies and dose estimation

**Fig. 2:**
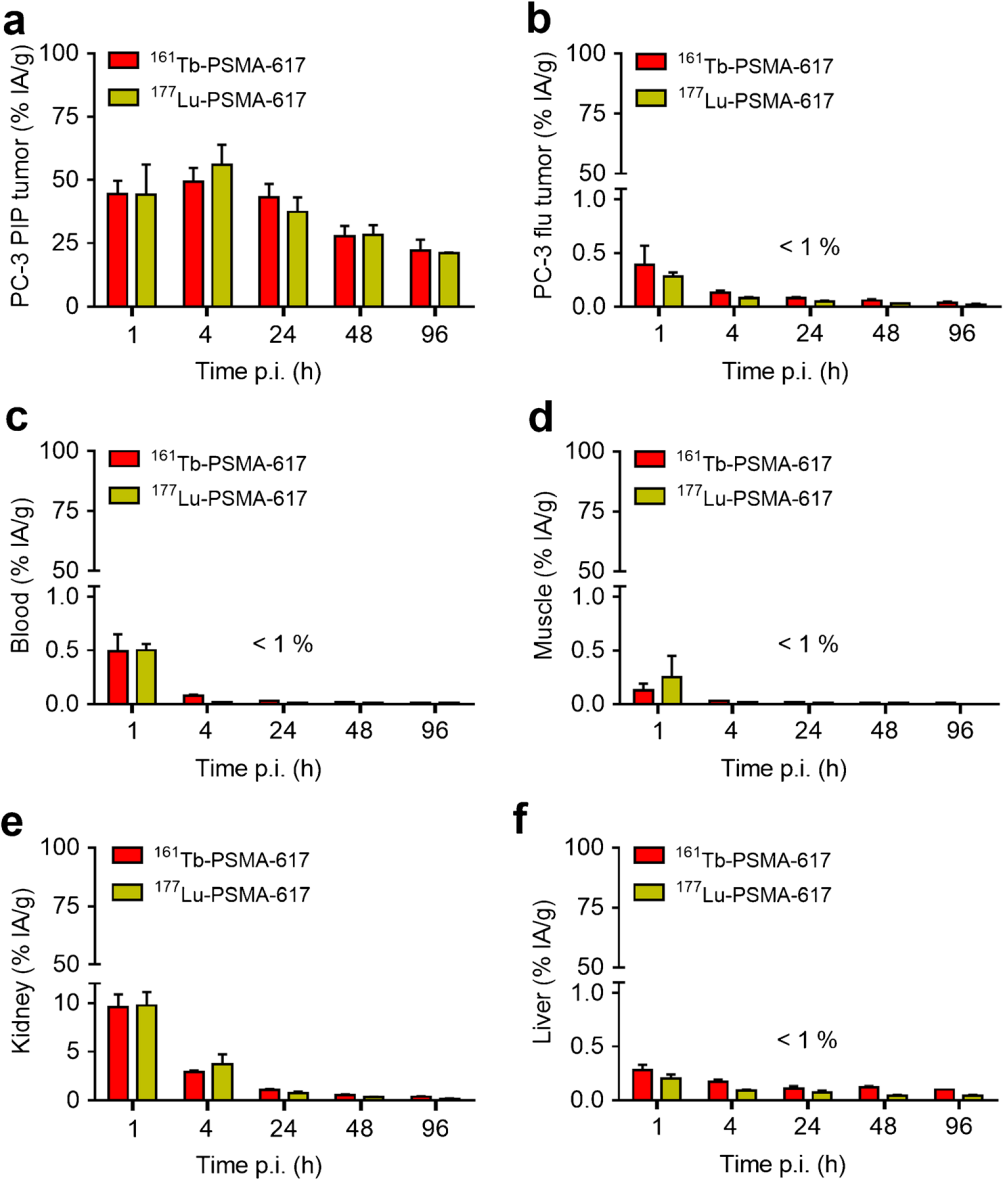
Biodistribution data of ^161^Tb-PSMA-617 in comparison to ^177^Lu-PSMA-617 in PC-3 PIP/flu tumor-bearing mice until 96 h p.i. (average ± SD, n = 3–5). **a** Uptake in PC-3 PIP tumors (PSMA positive). **b** Uptake in PC-3 flu tumors (PSMA negative). **c** Blood activity levels. **d** uptake in muscles. **e** Renal retention and **f** uptake in the liver.

Time-dependent biodistribution of ^161^Tb-PSMA-617 was assessed in PC-3 PIP/flu tumor-bearing mice and compared to the data previously obtained with ^177^Lu-PSMA-617 (Supplementary material, Table S2) [27, 32]. The uptake of ^161^Tb-PSMA-617 in PC-3 PIP tumor xenografts reached a maximum at 4 h p.i. (49 ± 5.5% IA/g) and decreased slowly over time (22 ± 4.3% IA/g at 96 h p.i.). Accumulation of ^161^Tb-PSMA-617 in PC-3 flu tumors and other non-targeted organs was in the range of blood activity levels or below at any evaluated time point. The radioligand was cleared via the kidneys over the first few hours after injection (9.6 ± 1.3% IA/g; 1 h p.i. 2.9 ± 0.14% IA/g; 4 h p.i.). These results confirmed that the tissue distribution profile of ^161^Tb-PSMA-617 was equal (*p* > 0.05) to the data previously published for ^177^Lu-PSMA-617 (Fig. 2) [27, 32].

For the absorbed dose estimations, the absorbed fractions of the assumed spherical tumors (80 mm^3^) were almost equal, with 0.96 and 0.93 for ^161^Tb and ^177^Lu respectively. The estimated mean specific absorbed dose to the PC-3 PIP tumors was 5.34 Gy/MBq and 3.90 Gy/MBq for ^161^Tb-PSMA-617 and ^177^Lu-PSMA-617 respectively. This resulted in 27 Gy (5.0 MBq/mouse) and 53 Gy (10 MBq/mouse) in mice treated with ^161^Tb-PSMA-617, and would result in 20 Gy (5.0 MBq/mouse) and 39 Gy (10 MBq/mouse) if mice were treated with ^177^Lu-PSMA-617. The mean specific absorbed dose to the kidneys for ^161^Tb and ^177^Lu was determined as 0.062 Gy/MBq and 0.045 Gy/MBq respectively, resulting in 0.31 Gy (5.0 MBq/mouse) and 0.62 Gy (10 MBq/mouse) in mice treated with ^161^Tb-PSMA-617. Should ^177^Lu-PSMA-617 be used, it would result in a kidney dose of 0.225 Gy (5.0 MBq/mouse) and 0.45 Gy (10 MBq/mouse) respectively.

### SPECT/CT imaging

**Fig. 3.**
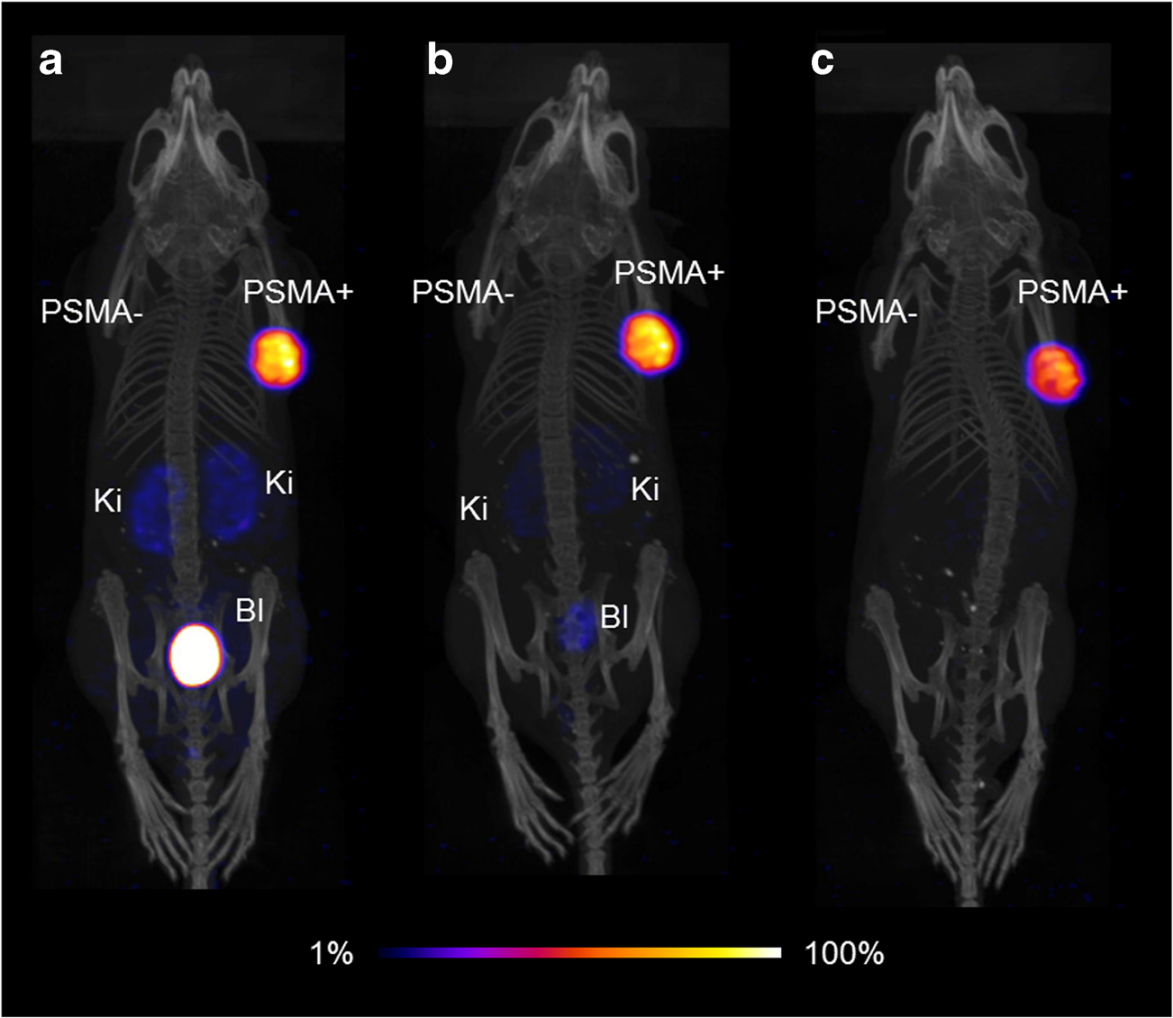
SPECT/CT images of mice after injection of ~25 MBq ^161^Tb-PSMA-617 shown as maximum intensity projections. **a** Scan obtained 1 h p.i.. **b** Scan obtained 4 h p.i.. **c** Scan obtained 24 h p.i..

SPECT/CT scans of PC-3 PIP/flu tumor-bearing mice were obtained at 1 h, 4 h, and 24 h after injection of ~25 MBq ^161^Tb-PSMA-617, resulting in images that were comparable to those previously obtained with ^177^Lu-PSMA-617 (Fig. 3) [27]. Radioligand accumulation was visualized in the PC-3 PIP tumor xenograft on the right side, while negligible uptake was seen in the PSMA-negative PC-3 flu tumor on the left side. Renal excretion of ^161^Tb-PSMA-617 was fast and the activity almost entirely excreted after 4 h.

### Preclinical tumor therapy

**Fig. 4:**
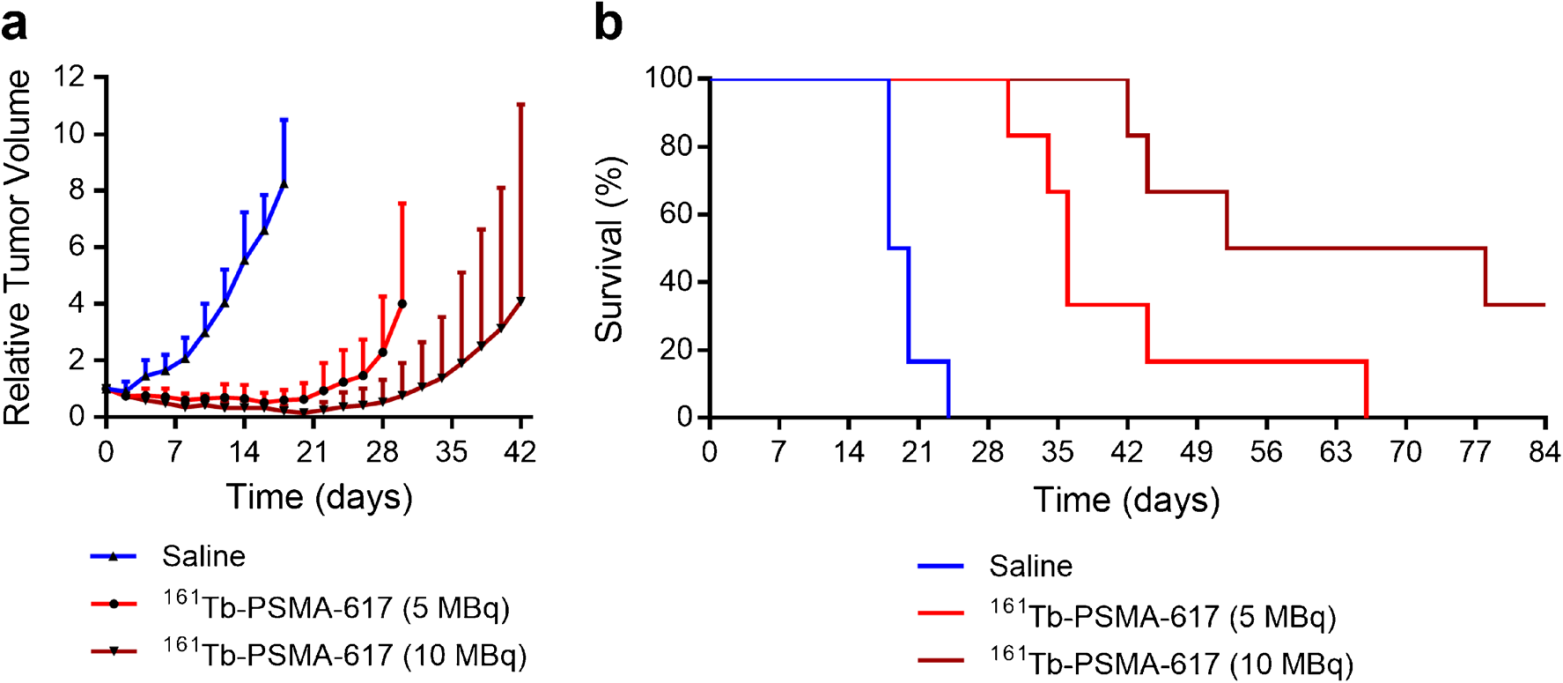
Graphs representing tumor growth and survival of control mice (*blue*) and mice treated with ^161^Tb-PSMA-617 (*red* and *dark red*) with each group comprising of *n* = 6 mice. **a** Average tumor size shown until the first mouse of each group reached an endpoint; untreated control mice (*blue*), mice injected with 5.0 MBq ^161^Tb-PSMA-617 (*red*) and mice injected with 10 MBq ^161^Tb-PSMA-617 (*dark red*). **b** Kaplan–Meier plot with survival curves of control mice (19 days), mice treated with 5.0 MBq ^161^Tb-PSMA-617 (36 days) and 10 MBq ^161^Tb-PSMA-617 (65 days)days), respectively.

**Fig. 5:**
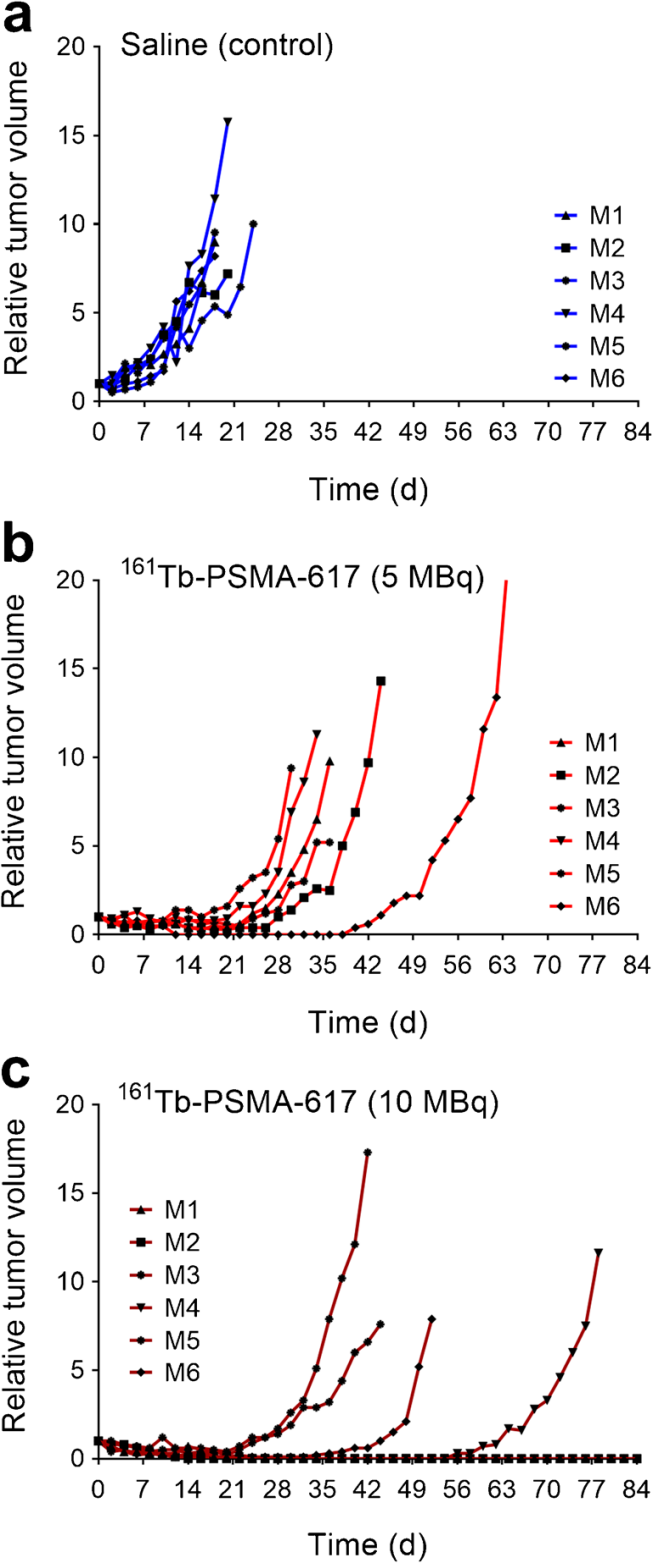
Graphs representing individual tumor growth of control mice (*blue*) and mice treated with ^161^Tb-PSMA-617 (*red* and *dark red*). **a** Individual mice injected with saline. **b** Individual mice treated with ^161^Tb-PSMA-617 (5.0 MBq/mouse). **c** Individual mice treated with ^161^Tb-PSMA-617 (10 MBq/mouse).

Constant tumor growth over time was observed in untreated mice of the control group, resulting in three mice that reached the endpoint at Day 18. The tumor growth of mice treated with 5.0 MBq and 10 MBq ^161^Tb-PSMA-617 was delayed, and hence the first mouse from these groups had to be euthanized at Day 30 and Day 42 respectively. The mice from the group treated with 5.0 MBq ^161^Tb-PSMA-617 were terminated when the last mouse of the group reached the endpoint at Day 66; however, in the group treated with 10 MBq ^161^Tb-PSMA-617, two mice were still alive at the end of the study at Day 84 (Table 2; Fig. 4). The tumor response in mice that received 10 MBq ^161^Tb-PSMA-617 was highly variable among the six mice, ranging from similar effects to those observed after injection of 5.0 MBq ^161^Tb-PSMA-617 to complete tumor remission (Fig. 5).

**Table 2:** Various parameters characterizing the efficacy of the treatment

Treatment	Injected activity	First mouse euthanized	Last mouse euthanized	Median survival	TGDI_2_	TGDI_3_
	(MBq)	(Day)	(Day)	(Day)		
Saline	–	18	24	19	1.0 ± 0.4	1.0 ± 0.1
^161^Tb-PSMA-617	5.0	30	66	36	4.2 ± 1.2	2.5 ± 0.6
^161^Tb-PSMA-617	10	42	84^1)^	65	n.d.	n.d.

^1^all mice were euthanized at the end of the study at Day 84 even though 2 mice had not reached an endpoint.

The median survival time of mice treated with ^161^Tb-PSMA-617 was 36 days, which was clearly longer than the median survival of the control mice (19 days). The application of 10 MBq ^161^Tb-PSMA-617 increased the median survival of mice to 65 days. In two of the six cases of this group, the PC-3 PIP tumors disappeared entirely, so that the mice survived over 12 weeks without any signs of tumor regrowth (Figs. 4 and 5).

### Monitoring of mice during therapy

In the group of mice that received 10 MBq ^161^Tb-PSMA-617, the body weight was slightly higher than in the other two groups at therapy start. While control mice and mice that received 5.0 MBq ^161^Tb-PSMA-617 experienced body weight loss over time, the body weight of mice that received 10 MBq ^161^Tb-PSMA-617 remained stable (Supplementary material, Table S3). In line with this result, the average absolute organ mass, calculated for kidney, liver and spleen of these mice, were also higher compared to those recorded in mice from the two other groups. The same held true for these organ masses calculated relative-to-body mass and relative-to-brain mass (Supplementary material, Table S3/S4). This indicates that exposure to ^161^Tb-PSMA-617 at 10 MBq per mouse mitigated the detrimental effects on the general health status observed in the other groups, which were probably caused by the rapidly growing tumors.

Evaluation of selected clinical chemistry parameters of renal and hepatic function (CRE, BUN ALP, TBIL, ALB) and the histological analysis of the bone marrow and salivary glands revealed no meaningful difference between the different groups (Supplementary material, Tables S5/S6).

### Additional investigations

A better tumor response to ^161^Tb-PSMA-617 treatment as compared to ^177^Lu-PSMA-617 was demonstrated in additional preclinical studies. In this case, mice received the radioligands already 2 days after PC-3 PIP tumor cell inoculation when the tumor tissue was not yet developed (Supplementary material; Table S7; Fig. S4). There was a clear trend of enhanced tumor growth inhibition and increased survival after application of ^161^Tb-PSMA-617 as compared to ^177^Lu-PSMA-617 at all activity levels (Supplementary material, Fig. S4/S5, Table S8).

## Discussion

In this study, ^161^Tb was investigated as a potential alternative to ^177^Lu to be used in combination with PSMA-targeting ligands. The production of no-carrier-added ^161^Tb has been developed to a quality that is comparable to that of no-carrier-added ^177^Lu, enabling efficient radiolabeling of biomolecules under the same experimental conditions. Attempts to label PSMA-617 with ^161^Tb at specific activities up to 100 MBq/nmol resulted in radiochemically pure ^161^Tb-PSMA-617 (> 98%). The radiolytic degradation of ^161^Tb-PSMA-617 was similar to ^177^Lu-PSMA-617, indicating that the emitted conversion and Auger electrons did not play a critical role with regard to the radioligand’s stability.

In agreement with previously-performed studies that compared ^161^Tb- and ^177^Lu-folate conjugates [29], the in-vitro properties of ^161^Tb-PSMA-617 and ^177^Lu-PSMA-617 were largely the same. This included the *n*-octanol/PBS distribution coefficient and cell uptake and internalization in PSMA-positive and PSMA-negative tumor cells. It was also confirmed that the pharmacokinetics of ^161^Tb-PSMA-617 was equal to ^177^Lu-PSMA-617, resulting in the same biodistribution profiles as expected (Fig. 2). It is likely that these findings can be extrapolated to any targeting agent with a DOTA-chelator; thus, ^161^Tb could replace ^177^Lu for any given biomolecule without changing its pharmacokinetic profile.

The enhanced therapeutic effects of ^161^Tb compared to ^177^Lu became obvious from in-vitro data where the exposure to ^161^Tb-PSMA-617 reduced the viability and survival of PC-3 PIP tumor cells in an activity-dependent manner. In agreement with dosimetric calculations, ^161^Tb-PSMA-617 was up to 3-fold more effective than ^177^Lu-PSMA-617 in vitro. This difference in efficacy of ^161^Tb-PSMA-617 and ^177^Lu-PSMA-617 was not observed when using PSMA-negative PC-3 flu cells or when PC-3 PIP cells were exposed to the DTPA-complexes of the two radionuclides. These findings confirmed that the observed advantage of using ^161^Tb-PSMA-617 over ^177^Lu-PSMA-617 is dependent on PSMA binding and internalization. The in-vitro findings also corroborated previous in-vitro findings, where ^161^Tb-folate was more effective in reducing KB tumor cell viability than ^177^Lu-folate [29].

The treatment of PC-3 PIP tumor-bearing mice with 5.0 MBq and 10 MBq ^161^Tb-PSMA-617, respectively, showed an activity-dependent tumor growth inhibition and prolonged survival of mice. When ^161^Tb-PSMA-617 was applied at 10 MBq, the tumor xenografts disappeared entirely in two out of six mice, which were still alive at study-end after 12 weeks. As no signs of undesired side-effects were detectable, higher activities may be used to treat the tumors more effectively. The tumor growth inhibition and median survival (TGDI_2_ = 4.2 ± 1.2; 36 days; Table 2) of mice that received 5.0 MBq ^161^Tb-PSMA-617 indicated better therapy response that that achieved in previously-reported results obtained with 5.0 MBq ^177^Lu-PSMA-617 (TGDI_2_ = 2.1 ± 0.3, median survival: 32 days [34]). Individual mice treated with 5.0 MBq ^161^Tb-PSMA-617 revealed a heterogeneous response pattern, where the last mouse reached the endpoint at Day 66. In contrast, the use of ^177^Lu-PSMA-617 therapy resulted in the last mouse to be euthanized at Day 40 [34].

In additional experiments, we simulated the situation of tumor cells in vivo that have not yet grown to a tissue, in order to investigate whether the radioligands delayed the formation of solid tumors (Supplementary material). At that time, a vascularized tissue was not yet developed, and the measurable “swelling” could presumably be ascribed to the formation of a tumor cell cluster. When applied at activities of 2.5 MBq, 5.0 MBq, or 10 MBq, the effect of ^161^Tb-PSMA-617 was enhanced when compared to that of ^177^Lu-PSMA-617, and re-growth of already disappeared tumors was less frequent when using ^161^Tb-PSMA-617 (Supplementary material; Fig. S4; Table S8). These results confirmed the anticipated improved effect of ^161^Tb over ^177^Lu also at the level of single cancer cells or cancer cell clusters in vivo.

In line with these results, the dosimetry analysis revealed that ^161^Tb has a 1.4-fold higher energy deposition in established tumors compared to ^177^Lu. This ratio increases to about 4-fold for small cell clusters and single cells. Together with the biological results obtained in this study, the dosimetry confirms that ^161^Tb may be better suited than ^177^Lu for sterilizing small cell clusters in advanced metastatic prostate cancer with radiolabeled PSMA ligands.

To date, it remains unclear to what extent the design of the targeting ligand could contribute to fully exploiting the decay properties of ^161^Tb. It has been stated in literature that nuclear localization is necessary to obtain effective Auger electron therapy [35–38]. In the case of ^161^Tb, the additional effect is, however, given predominantly by the emission of conversion electrons of an energy and tissue range comparable to β^¯^-particles of lowest energy. Hence, even when neglecting Auger electrons, the absorbed dose of ^161^Tb is still superior to that of ^177^Lu due to more emitted electrons per decay. It remains to be investigated whether PSMA ligands, comprising a nuclear localizing signal for effective delivery of the radionuclide to the cell nucleus, would improve the effect of ^161^Tb further by also making full use of the emitted Auger electrons. More sophisticated ligand designs and more clinically-relevant mouse models for testing the effects will be the topic of future preclinical studies to obtain answers to these open questions.

## Conclusion

^161^Tb was used for the first time with a PSMA ligand, which demonstrated better results than ^177^Lu-PSMA-617 in vitro and in vivo. Based on these findings, the postulated superiority of ^161^Tb over ^177^Lu was corroborated. Our preclinical research activities will be continued to further investigate ^161^Tb, as we intend to translate it to clinics and provide prostate cancer patients with an optimized treatment option in the near future.

## Electronic supplementary material

#### Associated content

Supplementary material comprises information about: the production of ^161^Tb, HPLC-based quality control of radioligands, stability experiments, logD values, cell internalization studies, methods of in-vitro assays and in-vitro dosimetry, tables of biodistribution data, dosimetry calculations, method of SPECT/CT imaging experiments, assessment of the therapy study, and additional therapeutic investigations.ESM 1(DOCX 1.93 mb)
