# Supplementary material for: Terbium-161 for PSMA-targeted radionuclide therapy of prostate cancer
Source: Eur J Nucl Med Mol Imaging. 2019 May 27;46(9):1919–30. doi: 10.1007/s00259-019-04345-0 (PMC6820371; doi:10.1007/s00259-019-04345-0)
Supplement: ESM 1 — (DOCX 1.93 mb) [file 259_2019_4345_MOESM1_ESM.docx]

**SUPPLEMENTARY MATERIAL**

**Terbium-161 for PSMA-targeted radionuclide therapy of prostate cancer**

Cristina Müller^1*^, Christoph A. Umbricht^1^, Nadezda Gracheva^1^, Viviane J. Tschan^1^, Giovanni Pellegrini^2^, Peter Bernhardt^3^, Jan Rijn Zeevaart^4^, Ulli Köster^5^, Roger Schibli^1,6^, Nicholas P. van der Meulen^,1,7^

1. Center for Radiopharmaceutical Sciences ETH-PSI-USZ, Paul Scherrer Institute, 5232 Villigen-PSI, Switzerland

2. Laboratory for Animal Model Pathology, Institute of Veterinary Pathology, Vetsuisse Faculty, University of Zurich, 8057 Zurich, Switzerland

3. Department of Radiation Physics, Institution of Clinical Science, Sahlgrenska Academy, University of Gothenburg, 413 45 Gothenburg, Sweden

4. Radiochemistry, South African Nuclear Energy Corporation (Necsa), 0240, Brits, South Africa

5. Institut Laue-Langevin, 38042 Grenoble, France

6. Department of Chemistry and Applied Biosciences, ETH Zurich, 8093 Zurich, Switzerland

7. Laboratory of Radiochemistry, Paul Scherrer Institute, 5232 Villigen-PSI, Switzerland

***Correspondence to**:

PD Dr. Cristina Müller

Center for Radiopharmaceutical Sciences ETH-PSI-USZ

Paul Scherrer Institut

5232 Villigen-PSI

Switzerland

e-mail: cristina.mueller@psi.ch

phone: +41-56-310 44 54; fax: +41-56-310 28 49

**1. Radionuclides**

**Purpose:** ^161^Tb was proposed as an alternative radiolanthanide to the clinically employed ^177^Lu, however, it is not yet commercially available. The production of ^161^Tb is currently only performed at PSI.

**Methods:** The production of ^161^Tb using the ^160^Gd(n,γ)^161^Gd→^161^Tb nuclear reaction was performed as previously reported [1]. Irradiations of enriched ^160^Gd_2_O_3_ targets (7‒32 mg ^160^Gd) were performed over a period of 1‒2 weeks in the high neutron flux reactors SAFARI-1 Necsa, Pelindaba, South Africa or RHF at Institut Laue-Langevin (ILL), Grenoble, France, in order to obtain 10‒20 GBq ^161^Tb. In some cases, 100 mg enriched ^160^Gd_2_O_3_ targets were irradiated at the spallation-induced neutron source SINQ (PSI) over a period of 3 weeks, which resulted in ~6 GBq ^161^Tb. Higher masses of the target material and longer irradiation periods were necessary for irradiations at SINQ due to the significantly lower neutron flux of the SINQ (~2·10^13^ n/cm^2^/s) as compared to the reactor neutron flux (SAFARI: 1.8·10^14^ n/cm^2^/s; ILL: 8·10^14^ n/cm^2^/s). ^161^Tb was separated from the Gd target material by cation exchange and extraction chromatography using an optimized process of the previously-reported procedure [1, 2].

**Results:** PSI-internal specifications were defined to release the produced ^161^Tb for experiments (Table S1). In terms of radiolabeling efficiency, the quality of ^161^Tb, formulated in 0.05 M HCl at high activity concentration, was comparable to the commercial no-carrier-added ^177^Lu. The determination of the identity of the product was performed using a high-purity germanium (HPGe) detector (Canberra, France) in combination with the InterWinner software package (version 7.1; Itech Instruments, France). The γ-spectrogram confirmed the presence of ^161^Tb-characteristic γ-lines. ^160^Tb was the only radionuclidic impurity, detected at levels <0.007% of total ^161^Tb activity at the end of the separation process. A detailed description of the production will be published elsewhere [3].

**Table S1** Specification of ^161^Tb produced at PSI

|  | **Specification** |
| --- | --- |
| **Activity concentration** | 10‒20 MBq/µL  ^161^Tb activity at end of separation |
| **Formulation** | HCl 0.05 M (suprapur) |
| **Appearance** | Clear and colorless solution |
| **Identity of ^161^Tb** | 48.9 keV γ-line  74.6 keV γ-line |
| **pH** | 1 – 2 |
| **Radionuclidic purity**  (γ-spectrometry) | ^160^Tb ≤ 0.007% of the total ^161^Tb activity  no other radionuclidic impurities detectable |
| **Radiochemical purity**  (radio-TLC) | ≥ 99% |
| **Radiolabeling yield**  HPLC, based on radiolabeling of DOTANOC with ^161^Tb at a molar ratio of 4:1 | ≥ 99% |

^177^Lu (no-carrier-added ^177^LuCl_3_ in 0.04 M HCl) was obtained from Isotope Technologies Garching, ITG GmbH, Garching, Germany.

Measurements of ^161^Tb and ^177^Lu activities for in-vitro and in-vivo experiments were performed using either the HPGe detector or a calibrated ionization chamber (Isomed 2010, Nuklear-Medizintechnik Dresden GmbH, Germany).

**2. Radiolabeling and quality control**

**Purpose:** Radiolabeling of PSMA-617 was performed under standard labeling conditions, followed by quality control using HPLC.

**Methods:** PSMA-617 (Advanced Biochemical Compounds, ABX GmbH, Radeberg, Germany) was dissolved in MilliQ water in order to obtain a 1 mM-stock solution that was kept in 50 µL samples in the freezer. The radiolabeling of PSMA-617 with ^161^Tb was performed as previously reported for ^177^Lu [4]. In brief, ^161^Tb was added to a mixture of sodium acetate (0.5 M, pH ~8) and HCl (0.05 M) containing PSMA-617 to obtain specific activities of up to 100 MBq/nmol. The reaction mixture (pH 4.5) was incubated for 10 min at 95 °C. Quality control of the radiolabeled PSMA-617 was performed with a Merck Hitachi LaChrom HPLC system, equipped with a D-7000 interface, a L-7200 autosampler, a radioactivity detector (LB 506 B from Berthold, Germany) and a L-7100 pump connected with a reversed-phase C18 column (5 µm, 150×4.6 mm, Xterra^TM^, MS, Waters, USA). The mobile phase consisted of 0.1% trifluoroacetic acid (Sigma-Aldrich, USA) in MilliQ water (A) and acetonitrile (VWR Chemicals, USA; HPLC-grade) (B) using a linear gradient of solvent A (95–20% over 15 min) in solvent B at a flow rate of 1 mL/min. An aliquot (~0.3 MBq) of the radiolabeling solution was diluted in 100 µL MilliQ water containing sodium diethylenetriamine pentaacetic acid (Na-DTPA; 50 µM) for analysis.

**Results:** A representative chromatogram of ^161^Tb-PSMA-617 showed the same picture as that obtained for ^177^Lu-PSMA-617 (Fig. S1). ^161^Tb-PSMA-617 and ^177^Lu-PSMA-617 were used for in-vitro and in-vivo experiments without further purification.


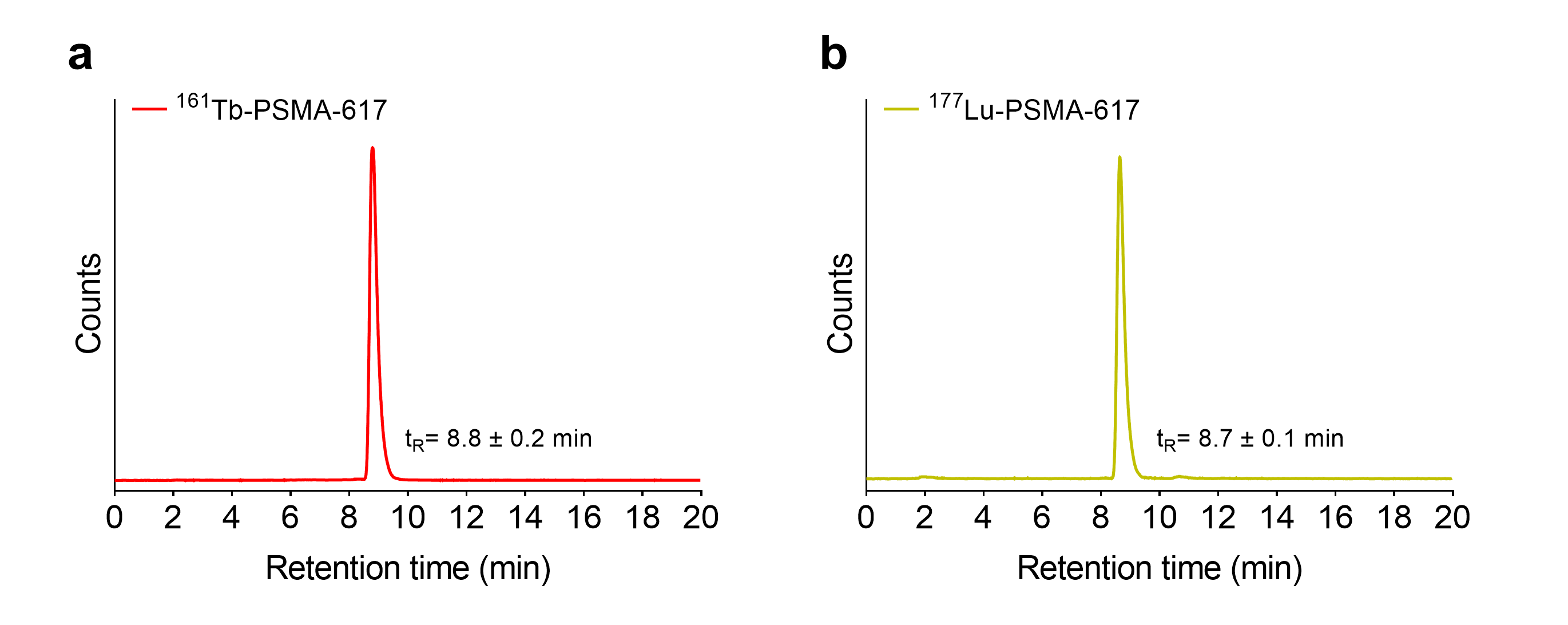


**Fig. S1** Representative HPLC chromatograms obtained after radiolabeling of PSMA-617 with ^161^Tb and ^177^Lu, respectively. (**a**) HPLC-based quality control of ^161^Tb-PSMA-617; (**b**) HPLC-based quality control of ^177^Lu-PSMA-617. Retention times are indicated in the graph (t_R_ = average retention time ± SD, n ≥6).

**3. Stability of ^161^Tb-PSMA-617 in comparison to ^177^Lu-PSMA-617**

**Purpose:** The stability of ^161^Tb-PSMA-617 incubated in saline at a high activity concentration (500 MBq/mL) was assessed over a period of 24 h for comparison with the previously-evaluated stability of ^177^Lu-PSMA-617 under the same experimental conditions [4].

**Methods:** PSMA-617 was labeled with ^161^Tb (50 MBq/nmol) in the absence or presence of l-ascorbic acid (3 mg) to investigate the radioligand’s in-vitro stability. After quality control using HPLC (t = 0, radiochemical purity ≥98%), the labeling solutions were diluted with saline (250 MBq in 500 µL) and incubated at room temperature. The integrity of ^161^Tb-PSMA-617 was determined using HPLC over time (t = 1 h, 4 h and 24 h, respectively), as previously reported [4]. The HPLC chromatograms were analyzed by integration of the peaks representing the radiolabeled product in relation to the sum of integrated peak areas of the entire chromatogram (set to 100%) including released ^161^Tb and degradation products of unknown composition.

**Results:** In the presence of l-ascorbic acid, ^161^Tb-PSMA-617 and ^177^Lu-PSMA-617 were entirely stable (≥98% intact radioligands) up to 24 h and did not show any signs of radiolytic degradation or release of the radiometal (Fig. S2).


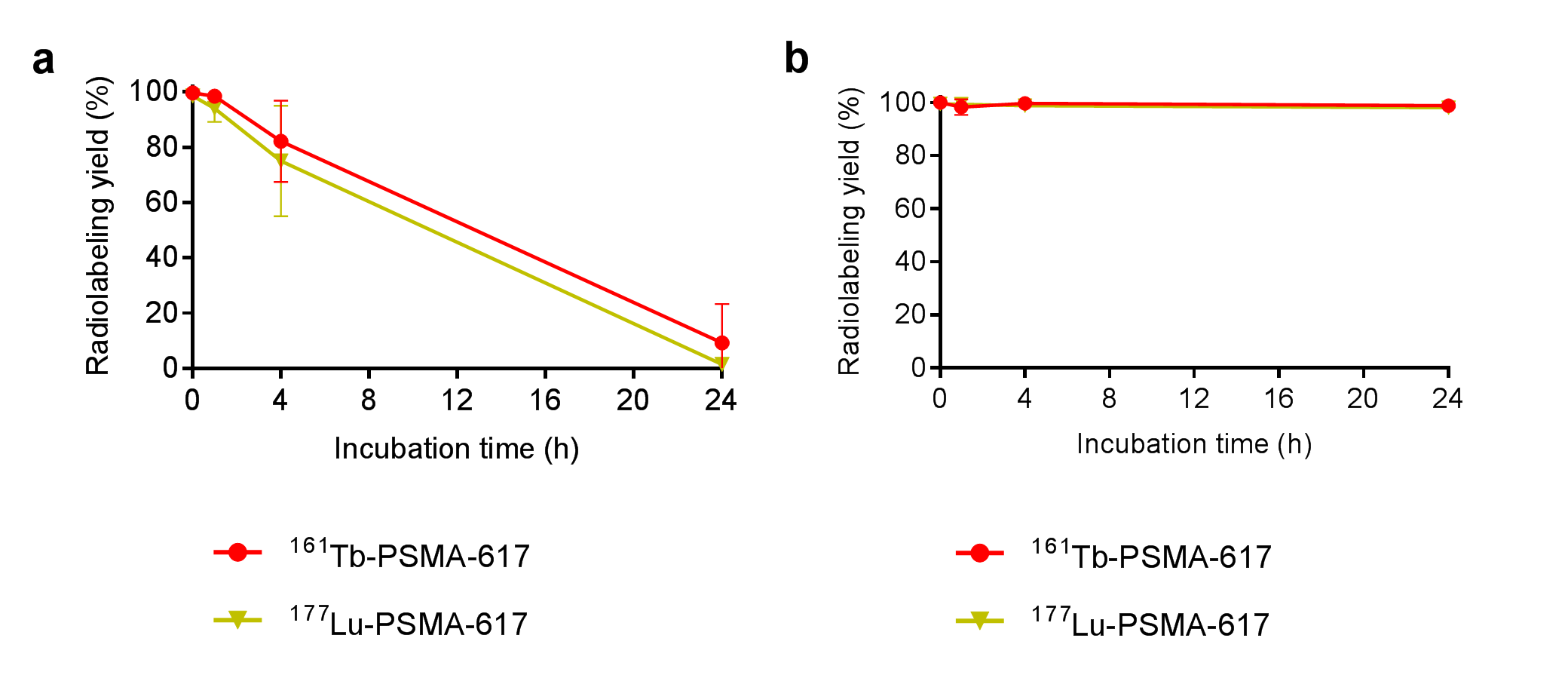


**Fig. S2** The graphs represent the percentage of intact ^161^Tb-PSMA-617 and ^177^Lu-PSMA-617 after incubation in saline with or without l-ascorbic acid. (**a**) Results obtained for samples of ^161^Tb-PSMA-617 (red) and ^177^Lu-PSMA-617 (yellow) incubated without l-ascorbic acid (average ± SD, n=3); (**b**) Results obtained for samples of ^161^Tb-PSMA-617 (red) and ^177^Lu-PSMA-617 (yellow) incubated with l-ascorbic acid (average ± SD, n=3). Data for ^177^Lu-PSMA-617 was obtained from Benešová et al. 2018 [4].

**4. Determination of *n*-octanol/PBS distribution coefficients (LogD values)**

**Purpose:** The distribution coefficient (logD values) of ^161^Tb-PSMA-617 was determined in order to confirm similar behavior with ^177^Lu-PSMA-617, as proposed.

**Methods:** The distribution coefficient (logD value) of ^161^Tb-PSMA-617 was determined in a 1:1 (*v/v*) mixture of *n*-octanol and phosphate-buffered saline (PBS pH 7.4) by a shake-flask method using liquid-liquid extraction followed by phase separation, as previously reported [5]. PSMA-617 was labeled with ^161^Tb at a specific activity of 50 MBq/nmol. It was diluted in PBS pH 7.4 (∼500 kBq, 25 µL, 0.01 nmol) and added to polypropylene tubes containing 1475 µL PBS and 1500 µL *n*-octanol followed by vigorous vortexing for 1 min. Phase separation was obtained by centrifugation at 1200 rcf for 6 min. The distribution coefficients were calculated as the logarithm of the ratio of counts per minute (cpm) measured in the *n*-octanol phase relative to the cpm measured in the PBS pH 7.4 phase using a γ-counter (Perkin Elmer, Wallac Wizard 1480). At least three experiments were performed in quintuplicate.

**Results:** The value of ^161^Tb-PSMA-617 was determined as –3.9 ± 0.1. This value was slightly higher than the previously determined value (–4.4 ± 0.1) for ^177^Lu-PSMA-617 by Benešová et al. [4].

**5. Cell culture**

**PC-3 PIP/flu tumor cells:** Sublines of the androgen-independent PC-3 human prostate cancer cell line, originally derived from an advanced androgen-independent bone metastasis, were kindly provided by Prof. Dr. Martin Pomper (John Hopkins Institutions, Baltimore, USA). The sublines had been created by transfection to express PSMA at high levels (PSMA^pos^ PC-3 PIP cells) or by mock transfection to obtain a cell line which does not express PSMA (PSMA^neg^ PC-3 flu cells) [6]. Both cell lines have been used by different groups, including our own, to evaluate PSMA-targeting radiopharmaceuticals [4, 5, 7-9]. These tumor cells were grown in a humidified incubator at 37 °C and 5% CO_2_ in RPMI-1640 cell culture medium (Bio Concept, Switzerland) supplemented with 10% fetal calf serum (Bio Concept, Switzerland), l-glutamine, antibiotics (Bio Concept, Switzerland) as well as puromycin (2 µg/mL; InvivoGen, USA) to maintain PSMA expression. Routine cell culture was performed twice a week using PBS/EDTA (2 mM) and trypsin/EDTA (Gibco, USA) for cell detachment.

**6. Tumor cell uptake and internalization studies**

**Purpose:** The purpose of this experiment was to demonstrate equal in-vitro properties of ^161^Tb-PSMA-617 and ^177^Lu-PSMA-617 with regard to the uptake and internalization into PSMA-positive tumor cells.

**Methods:** PC-3 PIP and PC-3 flu cells were seeded in 12-well plates (3 x 10^5^ cells in 2 mL medium/well) and incubated at 37 °C and 5% CO_2_ for adherence and growth overnight. Uptake and internalization of ^161^Tb-PSMA-617 in PC-3 PIP and PC-3 flu cells were determined as previously reported by Benešová et al. [4]. For this purpose, PSMA-617 was labeled with ^161^Tb or ^177^Lu at a specific activity of 50 MBq/nmol and added to each well containing 975 µL cell culture medium without supplements (∼37.5 kBq, 25 µL, 0.0075 nmol ligand). In all experiments, ^177^Lu-PSMA-617 was tested at the same time for comparison. The experiments were performed three times and in triplicate for both time points.

**Results:** Uptake of ^161^Tb-PSMA-617 in PC-3 PIP tumor cells was high after 2 h and 4 h incubation time (47% and 54%, respectively), while the internalized fraction was between 8% after 2 h and 11% after 4 h. These results were comparable to the uptake and internalization of ^177^Lu-PSMA-617 determined in parallel experiments (Fig. S3a). Uptake of the radioligands in PC-3 flu cells was below 0.5% (Fig. S3b).

**Conclusion:** Equal results for ^161^Tb- and ^177^Lu-labeled PSMA-617 were demonstrated in this study. These results were expected as it was shown previously that exchanging ^177^Lu with ^161^Tb did not influence the properties of small-molecular-weight targeting agents such as folate radioconjugates [10].

**
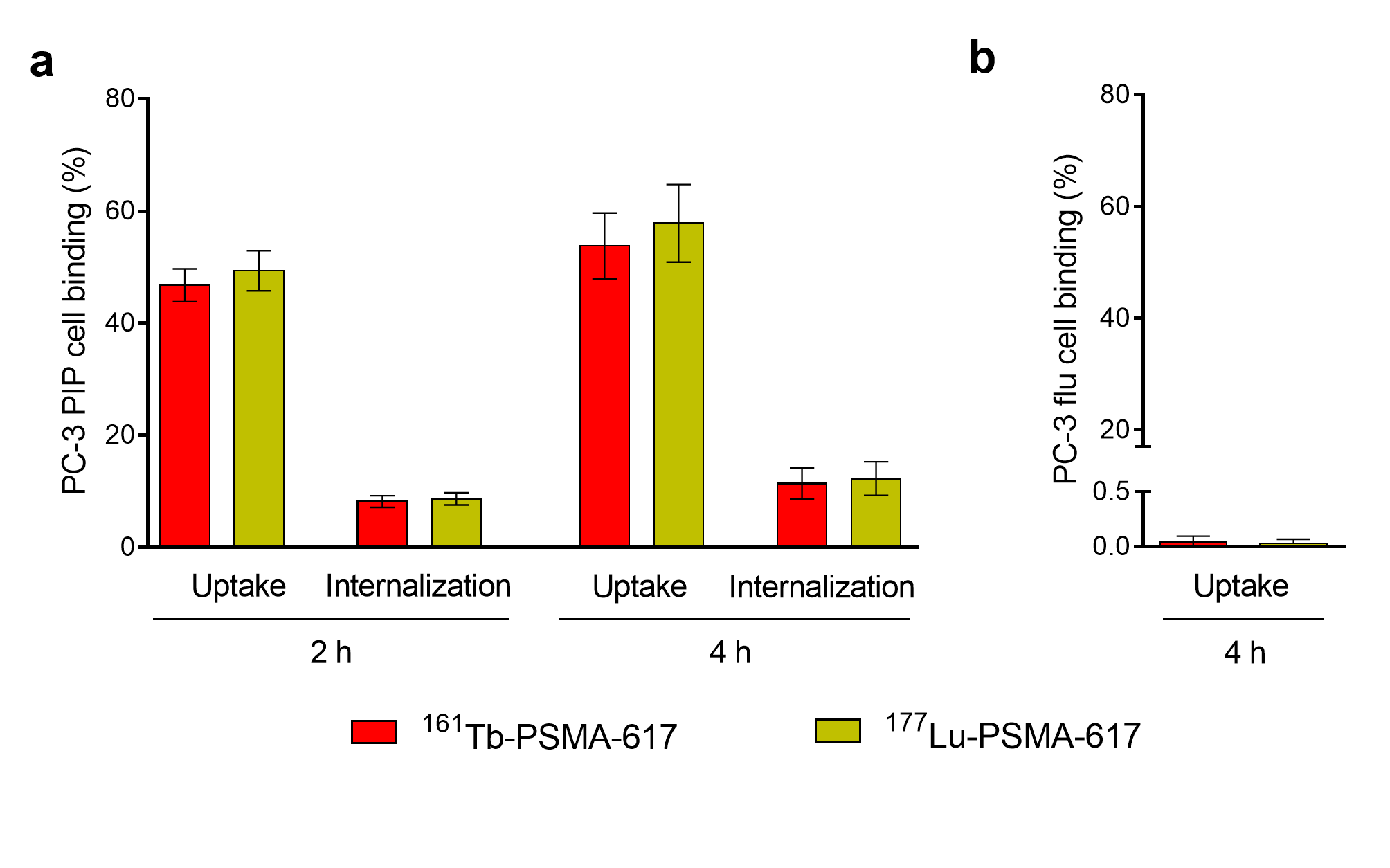
**

**Fig. S3** Cell uptake and internalization of ^161^Tb-PSMA-617 and ^177^Lu-PSMA-617 (average ± SD, n=3). (**a**) Data obtained with PSMA-positive PC-3 PIP tumor cells; (**b**) Data obtained with PSMA-negative PC-3 flu tumor cells.

**7. Cell viability assay (MTT assay)**

**Purpose:** Cell viability studies were performed using PC-3 PIP and PC-3 flu tumor cells to assess potentially different effects of ^161^Tb-PSMA-617 and ^177^Lu-PSMA-617.

**Methods:** Tumor cell viability was assessed using a 3-(4,5-dimethylthiazol-2-yl)-2,5-diphenyltetrazolium bromide (MTT) assay as described by Mosmann [11]. PC-3 PIP tumor cells (2500 cells in 200 μL RPMI medium with supplements) were seeded in 96-well plates and incubated overnight (37°C; 5% CO_2_) to allow adhesion of the tumor cells. The supernatants were removed and the tumor cells were washed once with 200 μL PBS. Afterwards, the tumor cells were incubated in 200 μL RPMI medium (without supplements) containing ^161^Tb-PSMA-617 or ^177^Lu-PSMA-617 (0.01‒20 MBq/mL), respectively, or just RPMI medium without addition of radioligands. PSMA-negative PC-3 flu tumor cells were also treated with these radioligands under the same experimental conditions using the same concentration range. Additional cell viability assays were performed with PC-3 PIP tumor cells incubated with RPMI medium (without supplements) containing ^161^Tb-diethylenetriaminepentaacetic acid (DPTA) or ^177^Lu-DTPA (0.01 – 20 MBq/mL). After 4 h incubation (37 °C; 5% CO_2_), the tumor cells were washed once with 200 μL PBS, followed by the addition of 200 μL of RPMI medium (with supplements) to each well. The tumor cells were left to grow for 6 days under standard cell culture conditions (37 °C, 5% CO_2_). At Day 6 after treatment, MTT reagent (5 mg/mL in PBS, 30 μL per well) was added to each well to allow the formation of dark-violet formazan crystals within 4 h incubation. The crystals were dissolved by the addition of dimethyl sulfoxide to determine the absorbance at 560 nm using a microplate reader (Victor X3, Perkin Elmer). Cell viability was quantified by expressing the absorbance of the test samples as percentage of the absorbance of control (untreated) cell samples which was set to 100%. Statistical analysis was performed using a two-way ANOVA with Sidak’s multiple comparison post-test in Graph Pad Prism (version 7).

**Results:** The results of these experiments are shown and discussed in the main manuscript.

**8. Cell survival assay (clonogenic assay)**

**Purpose:** Cell survival studies (clonogenic assay, also known as “colony forming assay”) were performed using PC-3 PIP and PC-3 flu tumor cells to assess potentially different effects of ^161^Tb-PSMA-617 and ^177^Lu-PSMA-617.

**Methods:** Tumor cell survival was evaluated using a clonogenic assay as described by Franken et al. [12]. PC-3 PIP tumor cells (200 cells in 2 mL RPMI medium with supplements) were seeded in 6-well plates and incubated overnight (37°C; 5% CO_2_) to allow adhesion of the tumor cells. The supernatants were removed and the tumor cells washed once with 1 mL PBS. Afterwards, the tumor cells were incubated in 1 mL RPMI medium (without supplements) containing ^161^Tb-PSMA-617 or ^177^Lu-PSMA-617 (0.01‒10 MBq/mL), respectively, or just RPMI medium without addition of radioligands. PSMA-negative PC-3 flu tumor cells were also treated with these radioligands under the same experimental conditions using the same concentration range. Additional cell viability assays were performed with PC-3 PIP tumor cells incubated with RPMI medium (without supplements) containing ^161^Tb-DPTA or ^177^Lu-DTPA (0.01 – 10 MBq/mL). After 4 h incubation (37 °C; 5% CO_2_), the tumor cells were washed once with 2 mL PBS followed by the addition of 2 mL RPMI medium (with supplements) to each well. The tumor cells were let to grow for 2 weeks under standard cell culture conditions (37 °C; 5% CO_2_). At Day 14 after treatment, the supernatants were removed and the tumor cells washed once with 1 mL PBS. The colonies were stained using crystal violet (0.5% crystal violet in 6.0% glutaraldehyde, 800 μL/well). All colonies of at least 0.5 mm diameter were counted manually as previously reported [13]. Cell survival was quantified by expressing the number of colonies of the test samples as percentage of the number of colonies of control (untreated) cell samples, which was set to 100%. Statistical analysis was performed using a two-way ANOVA with Sidak’s multiple comparison post-test in Graph Pad Prism (version 7).

**Results:** The results of the cell survival determined by clonogenic assays using PSMA radioligands are reported in the main manuscript.

**9. Dosimetry estimation for the cell viability and survival assays**

**Purpose:** The absorbed dose per decay to the adherent cells in the 96-well plates (MTT assay) and in the 6-well plates (clonogenic assay) was calculated in order to compare the estimated contribution of conversion and Auger electrons to the overall dose when using ^161^Tb-PSMA-617 as compared to ^177^Lu-PSMA-617.

**Method:** A model of one well in the 96-well plate was created and implemented into PENELOPE-2014 for Monte Carlo simulation of the absorbed energy/decay to cells, adherent to the bottom of the well. In the cell viability studies (MTT assay), 2500 cells were seeded per well using 96-well plates, which resulted in dispersed cells at the bottom of the wells. Some of these cells had large distances to neighboring cells, while others were attached to each other in small clusters. Based on this situation, the cells in the MTT experiment were exposed differently to cross-irradiation from neighboring cells. The two extreme situations were calculated in order to cover the whole range of different cross-irradiation situations. The one situation referred to a single cell located in the center of the well bottom (“single-cell-situation”), and in the other extreme, the whole surface area of the bottom (0.32 cm^2^) was covered with a monolayer of confluent cells (“monolayer-situation”). The volume of one cell was assumed to be 5110 µm^3^ [14]. The height of the monolayer was 11.1 µm. In the survival studies (clonogenic assay), 200 cells were seeded per well using 6-well plates, which resulted in separated single cells at the bottom of the well. In this situation, the cells were exposed as described above for the “single-cell-situation” in the MTT assay.

The total emitted mean energy from the emitted electrons and photons per decay for ^161^Tb and ^177^Lu (data from ([www.nucleide.org](http://www.nucleide.org)), was assessed by Monte Carlo simulations using PENELOPE-2014 [15].

**Results and Conclusions:** The mean absorbed energy per decay in the “single-cell situation” was 22.9 keV/decay and 5.4 keV/decay for ^161^Tb and ^177^Lu, respectively. The ^161^Tb/^177^Lu ratio between the absorbed energy per decay would then be 4.2. For a monolayer of confluent cells (“monolayer-situation”), the absorbed energy per decay in a cell would increase due to the cross-irradiation from neighboring cells. The mean absorbed energy per decay for the monolayer was 33.2 keV/decay and 10.3 keV/decay for ^161^Tb and ^177^Lu, respectively. The ^161^Tb/^177^Lu ratio between the absorbed energy per decay would then be 3.2.

In MTT assays, the tumor cells were not all exposed equally to cross-irradiation from neighboring cells and, hence, the ^161^Tb/^177^Lu energy per decay ratios varied between 3.2 and 4.2. The tumor cells in clonogenic assays were all irradiated in the “single-cell situation” and, hence, the ^161^Tb/^177^Lu ratio would be 4.2.

**10. Biodistribution studies**

**Purpose:** The purpose of the biodistribution studies performed in PC-3 PIP/flu tumor-bearing mice was to confirm equal pharmacokinetic properties of ^161^Tb-PSMA-617 as previously determined for ^177^Lu-PSMA-617 [4].

**Methods:** Female athymic nude BALB/c mice were obtained from Charles River Laboratories (Sulzfeld, Germany) at the age of ~5 weeks. Mice were inoculated with PSMA-positive PC-3 PIP tumor cells (6×10^6^ cells in 100 μL Hank’s balanced salt solution (HBSS) with Ca^2+^/Mg^2+^) on the right shoulder and PSMA-negative PC-3 flu tumor cells (5×10^6^ cells in 100 μL HBSS with Ca^2+^/Mg^2+^) on the left shoulder. Biodistribution studies were performed 12–14 days after tumor cell inoculation. The experimental procedure of the biodistribution studies is described in the main manuscript.

**Results:** The results of the tissue distribution of ^161^Tb-PSMA-617 in PC-3 PIP/flu tumor-bearing mice are given in Table S2.

**Table S2** Biodistribution of ^161^Tb-PSMA-617 in PC-3 PIP/flu tumor-bearing mice^1)^

|  | **^161^Tb-PSMA-617** | | | | |
| --- | --- | --- | --- | --- | --- |
| **Organ and tissues** | 1 h p.i. | 4 h p.i. | 24 h p.i. | 48 h p.i. | 96 h p.i. |
| Blood | 0.49 ± 0.16 | 0.08 ± 0.01 | 0.03 ± 0.00 | 0.02 ± 0.00 | 0.01 ± 0.00 |
| Heart | 0.21 ± 0.05 | 0.05 ± 0.01 | 0.03 ± 0.00 | 0.03 ± 0.00 | 0.02 ± 0.00 |
| Lung | 0.66 ± 0.28 | 0.11 ± 0.01 | 0.11 ± 0.09 | 0.04 ± 0.01 | 0.03 ± 0.00 |
| Spleen | 0.54 ± 0.08 | 0.13 ± 0.01 | 0.08 ± 0.01 | 0.07 ± 0.00 | 0.07 ± 0.01 |
| Kidneys | 9.6 ± 1.3 | 2.9 ± 0.14 | 1.1 ± 0.09 | 0.58 ± 0.05 | 0.40 ± 0.04 |
| Stomach | 0.22 ± 0.05 | 0.08 ± 0.01 | 0.04 ± 0.00 | 0.03 ± 0.00 | 0.66 ± 1.01 |
| Intestines | 0.26 ± 0.05 | 0.06 ± 0.01 | 0.03 ± 0.00 | 0.02 ± 0.00 | 0.12 ± 0.12 |
| Liver | 0.28 ± 0.05 | 0.17 ± 0.02 | 0.11 ± 0.02 | 0.12 ± 0.01 | 0.10 ± 0.00 |
| Muscle | 0.13 ± 0.06 | 0.03 ± 0.00 | 0.02 ± 0.00 | 0.01 ± 0.00 | 0.01 ± 0.00 |
| Bone | 0.21 ± 0.07 | 0.09 ± 0.02 | 0.09 ± 0.01 | 0.05 ± 0.01 | 0.06 ± 0.01 |
| Salivary glands | 0.28 ± 0.13 | 0.07 ± 0.01 | 0.05 ± 0.01 | 0.03 ± 0.00 | 0.03 ± 0.00 |
| PC-3 PIP tumor | 44 ± 5.3 | 49 ± 5.5 | 43 ± 5.5 | 28 ± 4.1 | 22 ± 4.3 |
| PC-3 flu tumor | 0.39 ± 0.18 | 0.13 ± 0.02 | 0.08 ± 0.01 | 0.06 ± 0.01 | 0.04 ± 0.01 |
| Tumor-to-blood | 99 ± 27 | 640 ± 132 | 1668 ± 302 | 1346 ± 166 | 2053 ± 412 |
| Tumor-to-liver | 163 ± 23 | 302 ± 64 | 383 ± 65 | 229 ± 30 | 224 ± 33 |
| Tumor-to-kidney | 4.7 ± 0.5 | 17 ± 1.6 | 43 ± 10 | 49 ± 7.2 | 56 ± 9.7 |

^1)^ The values are decay-corrected and presented as average %IA/g ± SD of values of n=3-5 mice.

**11. Dosimetry estimation for the in-vivo therapy**

**Purpose:** The absorbed dose to the tumors and kidney was calculated in order to compare the estimated contribution of Auger and conversion electrons to the overall dose when using ^161^Tb-PSMA-617 as compared to ^177^Lu-PSMA-617.

**Methods:** The mean specific absorbed doses (Gy/MBq) to the kidneys and the tumors were calculated as follows: The time-integrated activity, determined from created time-activity curves of non-decay corrected biodistribution data was multiplied with the total emitted mean electron energy of 197 keV/decay and 147 keV/decay, respectively, for ^161^Tb and ^177^Lu ([www.nucleide.org](http://www.nucleide.org)), the absorbed electron fractions for the kidney and tumors (assessed by Monte Carlo simulations using PENELOPE-2014) and a conversion factor. The time-activity curve for the kidney was obtained with a bi-exponential function, fitted to the non-decay corrected data points (R^2^>0.99). For the tumor, a linear function was used for the time points 0.5–8 h p.i. (R^2^>0.40) and a bi-exponential function for the time points 8–196 h p.i. (R^2^>0.99). The time-integrated activity was obtained by integration to infinity.

**Results:** In this therapy study, the mean size of the tumors 6 days after the subcutaneous inoculation of PC-3 PIP tumor cells was 80 mm^3^ (defined as Day 0; Table 1, main manuscript). The mean absorbed energy fraction calculated for this size of tumors was almost equal for ^161^Tb and ^177^Lu with 0.96 and 0.93, respectively. The estimated mean specific absorbed dose to these tumors was 5.34 Gy/MBq when using ^161^Tb-PSMA-617. In the case of using ^177^Lu-PSMA-617, the specific absorbed dose to the tumors would be 3.90 Gy/MBq. The mean absorbed dose to the tumors of mice of the therapy study was, therefore, 27 Gy and 53 Gy in mice treated with ^161^Tb-PSMA-617 (5.0 MBq/mouse and 10 MBq/mouse, respectively). In the case of using ^177^Lu-PSMA-617 the tumor dose would have been lower with 20 Gy and 39 Gy when mice would be treated with ^177^Lu-PSMA-617 (5.0 MBq/mouse and 10 MBq/mouse, respectively).

The mean specific absorbed dose to the kidneys for ^161^Tb and ^177^Lu was 0.062 Gy/MBq and 0.045 Gy/MBq, respectively. The mean absorbed dose to the kidneys of mice of the therapy study was, therefore, 0.31 Gy and 0.62 Gy, respectively, in mice treated with ^161^Tb-PSMA-617 (5.0 MBq/mouse and 10 MBq/mouse, respectively). If mice were treated with ^177^Lu-PSMA-617, the dose would be reduced to 0.225 Gy and 0.45 Gy for 5.0 MBq/mouse and 10 MBq/mouse, respectively.

**12. SPECT/CT imaging studies**

**Purpose:** SPECT/CT imaging studies were performed to visualize the accumulation of ^161^Tb-PSMA-617 in PC-3 PIP/flu tumor-bearing mice.

**Methods:** Mice were inoculated with PC-3 PIP/flu tumor cells, as described for biodistribution studies, and injected with ^161^Tb-PSMA-617 (~25 MBq, 1 nmol per mouse) about 12-14 days later. Under these conditions, the SPECT scans lasted for ~40 min. Lower activities could be used on the cost of longer acquisition times and/or loss of resolution. During the in-vivo scans, mice were anesthetized using a mixture of Isoflurane and oxygen.

SPECT/CT experiments were performed using a dedicated small-animal SPECT/CT camera (NanoSPECT/CT^TM^, Mediso Medical Imaging Systems, Budapest, Hungary) as previously reported [4, 10]. The images were acquired using Nucline software (version 1.02, Mediso Ltd., Budapest, Hungary). The real-time CT reconstruction used a cone-beam-filtered backprojection. The reconstruction of SPECT data was performed using HiSPECT software (version 1.4.3049, Scivis GmbH, Göttingen, Germany) using γ-energies of 47.7 keV (± 10%) and 74.6 keV (± 10%) for ^161^Tb. Data was post-processed using VivoQuant (version 3.0, inviCRO Imaging Services and Software, Boston USA). The lower scale of radioactivity was cut by 1% to make tumors and kidneys best visible.

**Results:** The results of the SPECT/CT experiments are shown in the main manuscript.

**13. Therapy study – Assessment of potential early side effects**

**Purpose:** The therapy study was performed to demonstrate the effect of ^161^Tb-PSMA-617 at two different activity levels (5.0 and 10 MBq/mouse) and to compare the effect of ^161^Tb-PSMA-617 with previously published results using ^177^Lu-PSMA-617 applied at the same quantity of activity (5.0 MBq/mouse) [9]. In order to assess potentially undesired early side effects, blood plasma parameters and organ/tissue masses were determined when an endpoint was reached or at the study end at Day 84.

**Methods:** The therapy was started at Day 0 by injecting mice with the radioligands, 6 days after PC-3 PIP tumor cell inoculation (4×10^6^ cells in HBSS with Ca^2+^/Mg^2+^) on the right shoulder. The therapy design is given in Table 1 of the main manuscript.

***Endpoint criteria:*** Mice were euthanized when a predefined endpoint criterion was reached or when the study was finalized at Day 84. Endpoint criteria were defined as (i) body weight loss of >15%, (ii) a tumor volume of >800 mm^3^ (iii) a combination of body weight loss of >10% and a tumor volume of >700 mm^3^ or (iv) signs of unease and pain or a combination thereof.

***Body and organ mass and mass ratios:*** As a measure to identify undesired side effects, body weights were compared at the day when the first control mouse had to be euthanized (Day 18; Table S3). The relative body weight (RBW) and the relative tumor volume (RTV) were calculated as stated in the main manuscript. Kidneys, liver, spleen and brain were collected and weighed at the day of euthanasia when an endpoint was reached or at the end of the study at Day 84 (Table S3). The organ mass-to-body mass and the organ-mass-to-brain-mass ratios were calculated for kidneys, liver and spleen (Table S4). The average organ masses of mice from each group were analyzed for significance using a one-way ANOVA test with a Tukey’s post correction (GraphPad Prism software, version 7). A *p*-value of <0.05 was considered as statistically significant.

***Blood plasma parameters:*** Blood was taken from the retrobulbar vein immediately before euthanasia for determination of blood plasma parameters. The values of creatinine (CRE), blood urea nitrogen (BUN), alkaline phosphatase (ALP), total bilirubin (BIL) and albumin (ALB) were determined in the plasma after centrifugation of the blood using a dry chemistry analyzer (DRI-CHEM 4000i, FUJIFILM, Japan) (Table S5). The average blood plasma parameters of each group were analyzed for significance using a one-way ANOVA test with a Tukey’s post correction (GraphPad Prism software, version 7). A *p*-value of <0.05 was considered as statistically significant.

***Necropsy, tissue processing and histological examination:*** After euthanasia and exsanguination, mice were dissected and, after a full macroscopic examination, sternum, femur and salivary glands were quickly removed and fixed in 4% neutral-buffered formalin (Formafix, Hittnau, Switzerland). After adequate fixation, femoral and sternal bones were decalcified in 10% ethylenediamine tetraacetic acid over a period of 5 days. Both bones and the salivary glands were subsequently trimmed, dehydrated through graded alcohols and routinely paraffin wax embedded. Sections (3–5 µm) were prepared, mounted on glass slides and routinely stained with hematoxylin and eosin (HE). Microscopic findings in the HE-stained livers were classified with standard pathological nomenclature and grading system [16].

**Results:**

***Organ mass and mass ratios:*** The average body weights of the different groups of mice were comparable at Day 0 with the only exception of the average body weight that was significantly (*p*<0.05) higher in the group of mice that received 10 MBq ^161^Tb-PSMA-617 as compared to mice that received 5.0 MBq ^161^Tb-PSMA-617 (Table 1 of the main manuscript). At Day 18, when the first mouse of the control group had to be euthanized, as well as at the study end at Day 84, the average body weight of mice treated with 10 MBq ^161^Tb-PSMA-617 was significantly higher than the average body weight of all other groups (*p*<0.05). In agreement with these findings, also the kidney, liver and spleen masses in mice that received 10 MBq ^161^Tb-PSMA-617 were significantly higher as compared to the organ masses of all other groups (*p*<0.05), while no statistical significance was detected for the brain mass (Table S3).

After euthanasia, kidneys, liver, spleen and brain were collected and weighed (Table S3) to calculate the organ mass-to-brain mass ratios (kidney-to-brain, liver-to-brain and spleen-to-brain; Table S4), which are discussed in the main manuscript.

**Table S3** Body weight and organ weight of mice of the therapy study and corresponding ratios

| Group (n=6) | Injected activity | Body weight  at Day 0 | Body weight  at Day 18^1)^ | Body weight  at Day of euthanasia^2)^ | Kidney mass^2)^ | Liver mass^2)^ | Brain mass^2)^ | Spleen  Mass^2)^ |
| --- | --- | --- | --- | --- | --- | --- | --- | --- |
|  | (MBq) | (g) | (g) | (g) | (mg) | (mg) | (mg) | (mg) |
| Saline | - | 17 ± 0.4 | 15 ± 0.5 | 15 ± 0.6 | 196 ± 12 | 712 ± 77 | 371 ± 15 | 56 ± 11 |
| ^161^Tb-PSMA-617 | 5.0 | 17 ± 1.2 | 17 ± 1.2 | 15 ± 1.7 | 208 ± 19 | 737 ± 143 | 371 ± 15 | 63 ± 13 |
| ^161^Tb-PSMA-617 | 10 | 18 ± 1.0*^3)^ | 19 ± 1.2*^4)^ | 19 ± 1.6*^5)^ | 258 ± 41*^5)^ | 994 ± 65*^5)^ | 402 ± 18 | 92 ± 22*^5)^ |

^1)^ First mouse of control group euthanized.

^2)^ Data obtained at the day of euthanasia when an endpoint criterion was reached or at the end of the study.

^3)^ At Day 0, the average body weight of mice injected with ^161^Tb-PSMA-617 (10 MBq/mouse) was significantly higher (*p*<0.05) than the average body weight of mice injected with lower activity of ^161^Tb-PSMA-617 (5.0 MBq/mouse).

^4)^ At Day 18, the average body weight of mice injected with ^161^Tb-PSMA-617 (10 MBq/mouse) was significantly higher (*p*<0.05) than the average body weight of all other groups.

^5)^ At the day of euthanasia or at the end of the study (Day 84) the average body weight, kidney mass, liver mass and spleen mass of mice injected with ^161^Tb-PSMA-617 (10 MBq/mouse) was significantly higher (p<0.05) than the average respective organ masses of all other groups.

* indicates significantly different values (*p*<0.05)

**Table S4** Organ mass-to-body mass and organ mass-to-brain mass ratios

|  |  | Organ mass-to-body mass ratios^1)^ | | |
| --- | --- | --- | --- | --- |
| Group  n = 6 | Injected activity (MBq) | Kidney-to-body ratio | Liver-to-body ratios | Spleen-to-body ratios |
| Saline | - | 0.013 ± 0.001 | 0.047 ± 0.004 | 0.004 ± 0.001 |
| ^161^Tb-PSMA-617 | 5.0 | 0.014 ± 0.001 | 0.048 ± 0.010 | 0.004 ± 0.001 |
| ^161^Tb-PSMA-617 | 10 | 0.014 ± 0.001 | 0.054 ± 0.002 | 0.005 ± 0.001 |
|  |  | Organ mass-to-brain mass ratios^1)^ | | |
| Group  n = 6 | Injected activity (MBq) | Kidney-to-brain ratio | Liver-to-brain ratios | Spleen-to-brain ratios |
| Saline | - | 0.53 ± 0.03 | 1.9 ± 0.70 | 0.15 ± 0.03 |
| ^161^Tb-PSMA-617 | 5.0 | 0.50 ± 0.11 | 1.8 ± 0.71 | 0.16 ± 0.05 |
| ^161^Tb-PSMA-617 | 10 | 0.64 ± 0.09^*2)^ | 2.5 ± 0.87 | 0.23 ± 0.05^*3)^ |

^1)^ Ratios calculated based on organ mass determined at the day of euthanasia when an endpoint criterion was reached or at the end of the study at Day 84.

^2)^ The kidney-to-brain ratio of mice injected with ^161^Tb-PSMA-617 (10 MBq/mouse) was significantly higher (p<0.05) than the ratio of mice injected with lower activity of ^161^Tb-PSMA-617 (5.0 MBq/mouse).

^3)^ The spleen-to-brain ratio of mice injected with ^161^Tb-PSMA-617 (10 MBq/mouse) was significantly higher (p<0.05) than the ratio of mice injected with reduced activity of ^161^Tb-PSMA-617 (5.0 MBq/mouse) or mice injected with saline.

* indicates significantly different values (*p*<0.05)

***Blood plasma parameters:*** Blood plasma parameters did not show any significant differences (*p*>0.05) among the control group (untreated mice) and mice treated with ^161^Tb-PSMA-617 (Table S5).

**Table S5** Blood plasma parameters of mice determined at the time of euthanasia when an endpoint was reached or at the study end

| Group (n=6) | Injected activity | CRE | BUN | ALP | TBIL | ALB |
| --- | --- | --- | --- | --- | --- | --- |
|  | (MBq) | (µmol/L) | (mmol/L) | (U/L) | (µmol/L) | (g/L) |
| Saline | - | <18 | 7.7 ± 1.2 | 54 ± 5 | 4 ± 1 | 22 ± 6 |
| ^161^Tb-PSMA-617 | 5.0 | <18 | 8.3 ± 0.50 | 44 ± 1 | 4 ± 1 | 21 ± 1 |
| ^161^Tb-PSMA-617 | 10 | <18 | 7.8 ± 1.3 | 54 ±15 | 4 ± 1 | 19 ± 1 |

***Histopathological observations:*** There were no histological changes (NHAIR) in the bone marrow and the salivary glands that could be attributed to the application of ^161^Tb-PSMA-617 (Table S6).

**Table S6** Histological assessment of selected tissues of the therapy study

|  |  | **Bone marrow sternum** | **Sternum (bone)** | **Bone marrow femur** | **Femur and tibia (bone)** | **Salivary glands^1)^** | **Mandibular lymph node** |
| --- | --- | --- | --- | --- | --- | --- | --- |
| Control | M1 | NHAIR | NHAIR | NHAIR | NHAIR | NHAIR | SH 1 |
|  | M2 | NHAIR | NHAIR | NHAIR | NHAIR | NHAIR | LH 1 |
|  | M3 | NHAIR | NHAIR | NHAIR | NHAIR | NHAIR | SH 1 |
|  | M4 | NHAIR | NHAIR | NHAIR | NHAIR | NHAIR | NHAIR |
|  | M5 | n.a. | NHAIR | NHAIR | NHAIR | NHAIR | NHAIR |
|  | M6 | n.a | NHAIR | NHAIR | NHAIR | NHAIR | SH 1 |
| ^161^Tb-PSMA-617 (5 MBq) | M1 | NHAIR | NHAIR | NHAIR | NHAIR | NHAIR | LH 1 |
|  | M2 | NHAIR | NHAIR | NHAIR | NHAIR | NHAIR | NHAIR |
|  | M3 | NHAIR | NHAIR | NHAIR | NHAIR | NHAIR | n.a. |
|  | M4 | NHAIR | NHAIR | NHAIR | NHAIR | NHAIR | NHAIR |
|  | M5 | NHAIR | NHAIR | NHAIR | NHAIR | NHAIR | SH 1 |
|  | M6 | NHAIR | NHAIR | NHAIR | NHAIR | NHAIR | NHAIR |
| ^161^Tb-PSMA-617 (10 MBq) | M1 | IC 1 | NHAIR | NHAIR | NHAIR | NHAIR | SH 2  LH 1 |
|  | M2 | n.a. | NHAIR | NHAIR | NHAIR | NHAIR | SH 1 |
|  | M3 | IC 1 | NHAIR | NHAIR | NHAIR | NHAIR | n.a. |
|  | M4 | NHAIR | NHAIR | NHAIR | NHAIR | NHAIR | LH 1 |
|  | M5 | NHAIR | NHAIR | NHAIR | NHAIR | NHAIR | SH 1 |
|  | M6 | NHAIR | NHAIR | NHAIR | NHAIR | NHAIR | NHAIR |

1) Salivary glands including mandibular, sublingual and parotid glands

NHAIR = no histological abnormality is recognized; n.a. = not available; IC = increased cellularity, myeloid lineage; SH = Sinus histiocytosis; LH = lymphoid hyperplasia. Standard toxicologic pathology nomenclature and grading system: 1: minimal severity; 2: mild; 3: moderate; 4: marked and 5: severe (grades 3-5 were not used).

**14. Additional investigations of therapeutic effects of ^161^Tb-PSMA-617**

**Purpose:** The purpose of these additional investigations was to assess potentially different effects of ^161^Tb-PSMA-617 and ^177^Lu-PSMA-617 on tumor cell growth when injected already 2 days after tumor cell inoculation. At this stage the tumor cells were already visible under the skin and measurable, however, not yet grown to a real tumor tissue.

**Methods:** The study was performed with mice inoculated with PC-3 PIP cells (4×10^6^ cells in HBSS with Ca^2+^/Mg^2+^) on the right shoulder 2 days before the treatment with the radioligands. Seven groups of mice with statistically similar body weights and tumor volumes were intravenously injected with only saline, ^161^Tb-PSMA-617 (2.5 MB/mouse, 5.0 MBq/mouse and 10 MBq/mouse, respectively) or ^177^Lu-PSMA-617 (2.5 MBq/mouse, 5.0 MBq/mouse and 10 MBq/mouse, respectively). The day of the treatment, two days after tumor cell inoculation, was defined as Day 0 of the study (Table S7).

**Table S7** Design of the Therapy Study B

| Treatment  (n=4) | Injected Radioactivity^1)^ | Tumor Volume^2)^ | Body Weight^2)^ |
| --- | --- | --- | --- |
|  |  | Day 0 | Day 0 |
|  | (MBq) | (mm^3^) | (g) |
| Saline | - | 77 ± 26 | 16 ± 1.8 |
| ^161^Tb-PSMA-617 | 2.5 | 63 ± 20 | 16 ± 1.6 |
| ^177^Lu-PSMA-617 | 2.5 | 52 ± 11 | 16 ± 1.2 |
| ^161^Tb-PSMA-617 | 5.0 | 72 ± 22 | 17 ± 1.1 |
| ^177^Lu-PSMA-617 | 5.0 | 67 ± 22 | 16 ± 1.2 |
| ^161^Tb-PSMA-617 | 10 | 70 ± 25 | 17 ± 0.7 |
| ^177^Lu-PSMA-617 | 10 | 85 ± 19 | 17 ± 0.8 |

^1^ The quantity of activity of the injection solutions for each group was confirmed by counting an injection sample (100 µL) using the dose calibrator.

^2^ No significant differences determined between the values measured for each group (*p*>0.05).

***Endpoint criteria:*** The mice were monitored until they reached an endpoint (definition of endpoints, see Chapter 13) or over the whole period of 38 days when the study was terminated.

***Data analysis of the therapy study:*** The tumor growth delay indices (TGDI_2_ and TGDI_5_) were calculated as reported in the Methods of the main manuscript. The median survival was determined using GraphPad Prism software (version 7). In the additional therapy study, the inoculated tumor cells were visible and, hence, measurable with a caliper.

***Dosimetry:*** At this early time point, it is, however, likely that the vasculature was not fully established and, hence, no solid tumor tissue was developed. It is assumed that the uptake of the radioligand in these tumor cells was much lower as compared to the uptake determined in biodistribution studies which were performed 12-14 days after tumor cell inoculation when small, vascularized tumors were developed. It was, therefore, not possible to perform dose estimations.

**Results:** The average tumor size of each group is shown in Fig. S4a. Tumor growth curves of individual mice are shown in Figs. S4b-S4h. Injection of only 2.5 MBq of the radioligands (Fig. S4c/d) did not delay tumor growth and showed similar results as in the case of untreated mice (Fig. S4b) reflected by similar values of the TGDI_2_ and TGDI_5_ (Table S8). At an activity of 5.0 MBq/mouse, the difference in effects between ^161^Tb-PSMA-617 and ^177^Lu-PSMA-617 became apparent by increased TGDI_2_ and TGDI_5_ in the group treated with ^161^Tb-PSMA-617 (Fig. S4e/f, Table S8). The different effects of ^161^Tb-PSMA-617 and ^177^Lu-PSMA-617 were impressively reflected in the groups of mice that received 10 MBq of the radioligands (Fig. S4g/h). In mice treated with ^177^Lu-PSMA-617 the tumors started to regrow in 50% of the cases from about Day 20 on (Fig. S4g). In the group of mice treated with ^161^Tb-PSMA-617, the tumors started to grow in only 2 of 8 cases from Day 25 and Day 35 on.

**
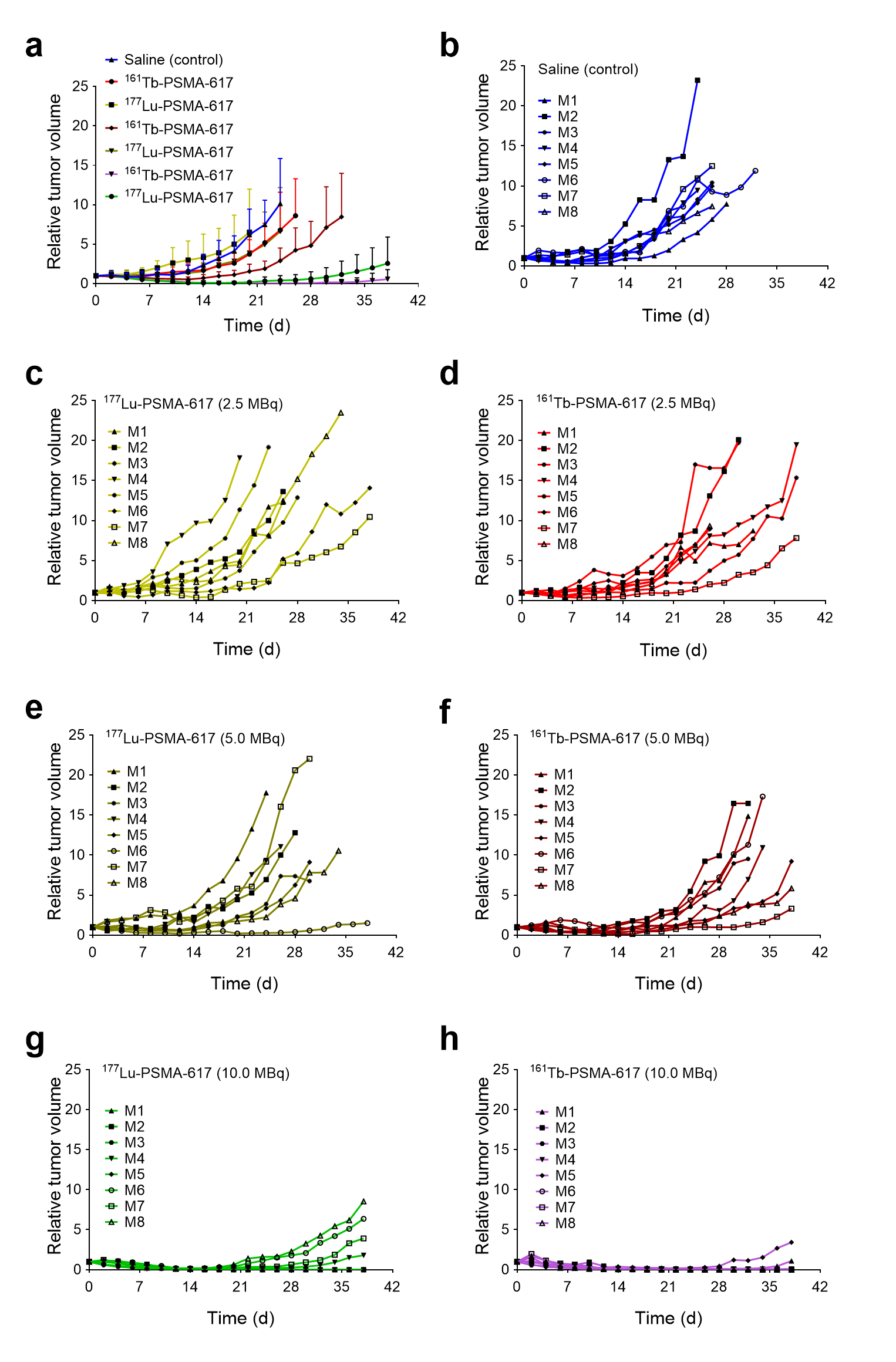
**

**Fig. S4** Graphs representing relative tumor growth of control mice (blue) and mice treated with different activities of ^161^Tb-PSMA-617 (red, dark red and violet) and ^177^Lu-PSMA-617 (yellow, dark yellow and green) two days after tumor cell inoculation (average ± SD, n=8). (**a)** Average relative tumor volumes of each group shown until the first mouse reached an endpoint; (**b**) Relative tumor volumes of individual mice injected with saline; (**c**) Relative tumor volumes of individual mice treated with ^161^Tb-PSMA-617 (2.5 MBq/mouse); (**d**) Relative tumor volumes of individual mice treated with ^177^Lu-PSMA-617 (2.5 MBq/mouse); (**e**) Relative tumor volumes of individual mice treated with ^161^Tb-PSMA-617 (5.0 MBq/mouse) and (**f**) Relative tumor volumes of individual mice treated with ^177^Lu-PSMA-617 (5.0 MBq/mouse). (**g**) Relative tumor volumes of individual mice treated with ^161^Tb-PSMA-617 (10 MBq/mouse). (**h**) Relative tumor volumes of individual mice treated with ^177^Lu-PSMA-617 (10 MBq/mouse).

The median survival of untreated control mice (26 d) as well as mice that received ^177^Lu-PSMA-617 (2.5 MBq/mouse) was almost the same (26 days and 27 days, respectively) while the treatment of ^161^Tb-PSMA-617 (2.5 MBq/mouse) resulted in a median survival time of 30 days. Injection of the radioligands at 5.0 MBq/mouse resulted also in an increased median survival of 34 days for ^161^Tb-PSMA-617 treated mice as compared to 30 days in the case of ^177^Lu-PSMA-617 treated mice. The groups that received 10 MBq ^161^Tb-PSMA-617 or 10 MBq ^177^Lu-PSMA-617 survived until the end of the study at Day 38 (Fig. S5 and Table S8).


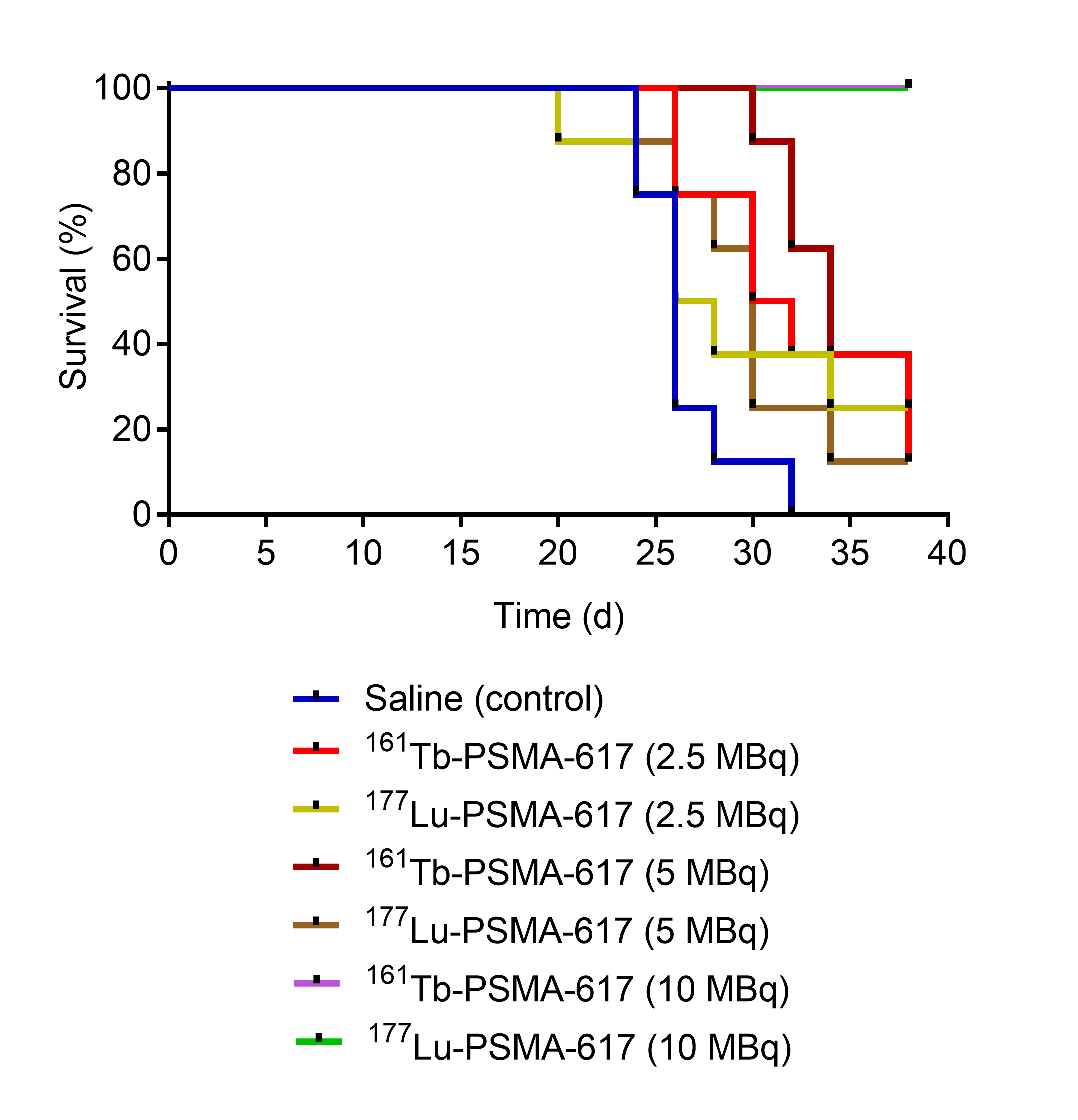


**Fig. S5** Kaplan-Meier plot with survival curves of mice of each group (n= 8). The median survival of control mice (blue) was 26 days. The median survival of mice treated with 2.5 MBq ^161^Tb-PSMA-617 (red) was 31 days but only 27 days for mice treated with 2.5 MBq ^177^Lu-PSMA-617 (yellow). The median survival of mice treated with 5.0 MBq ^161^Tb-PSMA-617 (dark red) was 34 days and, but only 30 days for those treated with 5.0 MBq ^177^Lu-PSMA-617 (brown). The median survival of mice treated with 10 MBq ^161^Tb-PSMA-617 (violet) and for mice treated with 10 MBq ^177^Lu-PSMA-617 (green) remained undefined as none of the mice reached an endpoint within the 38 day-period of this study.

The extent of tumor growth inhibition was also reflected by the values calculated for the TGDI_2_ and TGDI_5_, respectively (Table S8).

**Table S8** Various parameters characterizing the efficacy of the treatment

| **Treatment** | **Injected activity** | **First mouse euthanized** | **Last mouse euthanized** | **Median Survival** | **TGDI_2_** | **TGDI_5_** |
| --- | --- | --- | --- | --- | --- | --- |
|  | (MBq) | (Day) | (Day) | (Day) |  |  |
| Saline | - | 24 | 32 | 26 | 1.0 ± 0.3 | 1.0 ± 0.2 |
| ^161^Tb-PSMA-617 | 2.5 | 26 | study end | 31 | 1.0 ± 0.4 | 1.2 ± 0.3 |
| ^177^Lu-PSMA-617 | 2.5 | 20 | study end | 27 | 0.8 ± 0.4 | 1.0 ± 0.3 |
| ^161^Tb-PSMA-617 | 5.0 | 30 | study end | 34 | 1.6 ± 0.4 | n.d. |
| ^177^Lu-PSMA-617 | 5.0 | 24 | study end | 30 | 1.0 ± 0.4 | n.d. |
| ^161^Tb-PSMA-617 | 10 | study end | study end | n.d. | n.d. | n.d. |
| ^177^Lu-PSMA-617 | 10 | Study end | study end | n.d. | n.d. | n.d. |

n.d. = not determined since all mice were still alive at the end of the study or a 2-fold or 5-fold increase of the RTV was not reached for some of the mice of this groups.

**References**

1. Lehenberger S, Barkhausen C, Cohrs S, Fischer E, Grünberg J, Hohn A, et al. The low-energy beta^-^ and electron emitter ^161^Tb as an alternative to ^177^Lu for targeted radionuclide therapy. Nucl Med Biol. 2011;38:917-24. doi:S0969-8051(11)00044-8 [pii] 10.1016/j.nucmedbio.2011.02.007.

2. Müller C, Zhernosekov K, Köster U, Johnston K, Dorrer H, Hohn A, et al. A unique matched quadruplet of terbium radioisotopes for PET and SPECT and for α- and β¯-radionuclide therapy: An in vivo proof-of-concept study with a new receptor-targeted folate derivative. J Nucl Med. 2012;53:1951-9. doi:10.2967/jnumed.112.107540 jnumed.112.107540 [pii].

3. Gracheva N, Müller C, Talip Z, Heinitz S, Koester U, Zeevaart JR, et al. Production and characterization of no-carrier-added ^161^Tb as an alternative to the clinically-applied ^177^Lu for radionuclide therapy EJNMMI Radiopharmacy and Chemistry. 2019;submitted.

4. Benesova M, Umbricht CA, Schibli R, Müller C. Albumin-binding PSMA ligands: optimization of the tissue distribution profile. Mol Pharm. 2018;15:934-46. doi:10.1021/acs.molpharmaceut.7b00877.

5. Umbricht CA, Benesova M, Schmid RM, Türler A, Schibli R, van der Meulen NP, et al. ^44^Sc-PSMA-617 for radiotheragnostics in tandem with ^177^Lu-PSMA-617-preclinical investigations in comparison with ^68^Ga-PSMA-11 and ^68^Ga-PSMA-617. EJNMMI Res. 2017;7:9. doi:10.1186/s13550-017-0257-4.

6. Wu P, Kudrolli TA, Chowdhury WH, Liu MM, Rodriguez R, Lupold SE. Adenovirus targeting to prostate-specific membrane antigen through virus-displayed, semirandom peptide library screening. Cancer Res. 2010;70:9549-53. doi:10.1158/0008-5472.CAN-10-1760.

7. Banerjee SR, Pullambhatla M, Byun Y, Nimmagadda S, Green G, Fox JJ, et al. ^68^Ga-labeled inhibitors of prostate-specific membrane antigen (PSMA) for imaging prostate cancer. J Med Chem. 2010;53:5333-41. doi:10.1021/jm100623e.

8. Banerjee SR, Pullambhatla M, Foss CA, Nimmagadda S, Ferdani R, Anderson CJ, et al. ^64^Cu-labeled inhibitors of prostate-specific membrane antigen for PET imaging of prostate cancer. J Med Chem. 2014;57:2657-69. doi:10.1021/jm401921j.

9. Umbricht CA, Benesova M, Schibli R, Müller C. Preclinical development of novel PSMA-targeting radioligands: modulation of albumin-binding properties to improve prostate cancer therapy. Mol Pharm. 2018;15:2297-306. doi:10.1021/acs.molpharmaceut.8b00152.

10. Müller C, Reber J, Haller S, Dorrer H, Bernhardt P, Zhernosekov K, et al. Direct in vitro and in vivo comparison of ^161^Tb and ^177^Lu using a tumour-targeting folate conjugate. Eur J Nucl Med Mol Imaging. 2014;41:476-85. doi:10.1007/s00259-013-2563-z.

11. Mosmann T. Rapid colorimetric assay for cellular growth and survival: application to proliferation and cytotoxicity assays. J Immunol Methods. 1983;65:55-63.

12. Franken NA, Rodermond HM, Stap J, Haveman J, van Bree C. Clonogenic assay of cells in vitro. Nat Protoc. 2006;1:2315-9. doi:10.1038/nprot.2006.339.

13. Reber J, Haller S, Leamon CP, Müller C. ^177^Lu-EC0800 combined with the antifolate pemetrexed: preclinical pilot study of folate receptor targeted radionuclide tumor therapy. Mol Cancer Ther. 2013;12:2436-45. doi:10.1158/1535-7163.MCT-13-0422-T.

14. Yoshimori T, Takamatsu H. 3-D measurement of osmotic dehydration of isolated and adhered PC-3 cells. Cryobiology. 2009;58:52-61. doi:10.1016/j.cryobiol.2008.10.128.

15. Salvat F. PENELOPE-2014: A code system for monte carlo simulation of electron and photon transport. OECD/NEA Data Bank NEA/NSC/DOC. 2015;3:<http://www.nea.fr/lists/penelope.html>.

16. Shackelford C, Long G, Wolf J, Okerberg C, Herbert R. Qualitative and quantitative analysis of nonneoplastic lesions in toxicology studies. Toxicol Pathol. 2002;30:93-6. doi:10.1080/01926230252824761.
